# *Candidatus* Mycoplasma girerdii replicates, diversifies, and co-occurs with *Trichomonas vaginalis* in the oral cavity of a premature infant

**DOI:** 10.1038/s41598-017-03821-7

**Published:** 2017-06-19

**Authors:** Elizabeth K. Costello, Christine L. Sun, Erica M. Carlisle, Michael J. Morowitz, Jillian F. Banfield, David A. Relman

**Affiliations:** 1 Department of Medicine, Division of Infectious Diseases, Stanford University School of Medicine, Stanford, CA 94305 USA; 2 Department of Microbiology & Immunology, Stanford University School of Medicine, Stanford, CA 94305 USA; 3 Department of Surgery, Division of Pediatric Surgery, University of Iowa College of Medicine, Iowa City, IA 52242 USA; 4 Department of Surgery, Division of Pediatric Surgery, University of Pittsburgh School of Medicine, Pittsburgh, PA 15224 USA; 5 Department of Earth & Planetary Science, University of California, Berkeley, CA 94720 USA; 6 Veterans Affairs Palo Alto Health Care System, Palo Alto, CA 94304 USA

## Abstract

Genital mycoplasmas, which can be vertically transmitted, have been implicated in preterm birth, neonatal infections, and chronic lung disease of prematurity. Our prior work uncovered 16S rRNA genes belonging to a novel, as-yet-uncultivated mycoplasma (lineage ‘Mnola’) in the oral cavity of a premature neonate. Here, we characterize the organism’s associated community, growth status, metabolic potential, and population diversity. Sequencing of genomic DNA from the infant’s saliva yielded 1.44 Gbp of high-quality, non-human read data, from which we recovered three essentially complete (including ‘Mnola’) and three partial draft genomes (including *Trichomonas vaginalis*). The completed 629,409-bp ‘Mnola’ genome (*Candidatus* Mycoplasma girerdii str. UC-B3) was distinct at the strain level from its closest relative, vaginally-derived *Ca*. M. girerdii str. VCU-M1, which is also associated with *T*. *vaginalis*. Replication rate measurements indicated growth of str. UC-B3 within the infant. Genes encoding surface-associated proteins and restriction-modification systems were especially diverse within and between strains. In UC-B3, the population genetic underpinnings of phase variable expression were evident *in vivo*. Unique among mycoplasmas, *Ca*. M. girerdii encodes pyruvate-ferredoxin oxidoreductase and may be sensitive to metronidazole. This study reveals a metabolically unique mycoplasma colonizing a premature neonate, and establishes the value of genome-resolved metagenomics in tracking phase variation.

## Introduction

*Mycoplasma* species span three clades within the class Mollicutes, a group of obligate parasitic bacteria, affiliated with the phylum *Firmicutes*, that lack a cell wall. They establish relationships as commensals and pathogens of vertebrate hosts; in humans, colonization is typically limited to the mucosal surfaces of the respiratory and urogenital tracts. Invasive or disseminated infections do occur, but are more likely in immunocompromised or instrumented patients. Mycoplasmas are typically extracellular and surface-attached, but a few species can enter host cells and establish an intracellular niche. They evade the host immune system, in part by varying the expression of diverse surface proteins^1, 2^. Infections are often chronic, with untoward host immune responses contributing to the pathology^3, 4^.

Mycoplasmas are characterized by small cells, small genomes, and metabolic dependence on other organisms—qualities increasingly recognized as widespread across the tree of life^5–7^. AT-biased base composition and the use of genetic code four^8^ further distinguish the genomes. *De novo* biosynthesis of nucleotides, amino acids and fatty acids is minimal to absent in mycoplasmas, and energy metabolism is restricted to glycolysis, arginine hydrolysis or urea hydrolysis. Reputed as fastidious and slow-growing, generation times in culture range from ~1 hr for *Ureaplasma* spp., to 3 hr for *M*. *mycoides*, 6 hr for *M*. *pneumoniae*, and 16 hr for *M*. *genitalium* ^3, 4^. Long recognized as minimal self-replicating organisms, mycoplasmas and their study have sparked advances in genomics, synthetic biology, and the concept of a minimal cell^3, 9^.

Intrauterine infection is a major cause of preterm birth and mollicutes are the most frequently reported organisms in the amniotic cavity^10^. Among spontaneous preterm births, the earlier the gestational age at delivery, the more likely it is to be associated with intrauterine infection^11^. Vertical transmission is common, and in newborns, mycoplasmas and ureaplasmas can cause bacteremia, meningitis and pneumonia. *In utero* exposure to *Ureaplasma* spp. can also induce inflammatory responses leading to chronic lung injury in infants^12^. Infection control in the neonatal intensive care unit (NICU) has traditionally relied on culture-based surveillance. Given the fastidiousness of some mycoplasmas, and the expanding use of molecular surveillance methods, it is perhaps surprising that until recently^13^ no new species had been discovered in this setting^4^. One caveat is that cultivation-independent surveys of premature neonates have overwhelmingly prioritized the gut over sites more likely to harbor mycoplasmas such as the respiratory tract.

Several recent studies have characterized the vaginal microbiotas of women with trichomoniasis^14–16^. In one, Martin *et al*.^15^ reported a nearly full-length 16S rRNA gene sequence from an uncultivated *Mycoplasma* sp. (lineage ‘Mnola’) that was 85% identical to the closest human-associated species (*M*. *genitalium*, *M*. *pneumoniae*) and 94% identical to the closest cloned sequences, which are from cattle rumen and termite gut. These authors found ‘Mnola’ in 19/30 *T*. *vaginalis*-infected and 1/29 uninfected women attending a New Orleans, LA, sexually transmitted infection (STI) clinic^15^. In another study, Fettweis *et al*.^16^ detected *T*. *vaginalis* in 49/51 women whose vaginal microbiotas comprised at least 1% ‘Mnola’. These authors also reported a genome sequence for ‘Mnola’ (618,983-bp *Candidatus* Mycoplasma girerdii str. VCU-M1), which they assembled from the vaginal metagenome of a *T*. *vaginalis*-positive woman in preterm labor^16^. Although the number of observations is small, the presence of *Ca*. M. girerdii appears to indicate the presence of *T*. *vaginalis*, the most prevalent non-viral, sexually transmitted pathogen worldwide, and itself a risk factor for preterm birth^10, 17^. The presence of *T*. *vaginalis* does not necessarily indicate the presence of *Ca*. M. girerdii.

We uncovered abundant *Ca*. M. girerdii-like 16S rRNA gene sequences in the oral cavity of a vaginally-delivered premature neonate^13^. To date and to our knowledge, this remains the sole report of ‘Mnola’ in an infant; other than this case, this phylogenetically novel, as-yet-uncultivated mycoplasma has not been detected outside of vaginal samples. Here, we applied genome-resolved metagenomics to further examine the oral samples collected from the infant. Using this approach, we characterized the associated microbial community, growth status, metabolic potential, and within-population diversity of the infant-associated strain. Furthermore, the availability of the vaginally-derived genome (that of str. VCU-M1) provided a unique opportunity to compare the genomes of two closely related populations sampled directly from different host individuals.

## Methods

### Human subject, samples, and sequencing

Clinical details about the subject, a premature male infant, appear elsewhere (baby #3 therein)^13^. In brief, the infant was delivered vaginally, weighing 750 grams, at 24 weeks and 4 days of completed gestation. Preterm premature rupture of membranes (PPROM) complicated the delivery. As noted in the infant’s chart, the mother received prenatal care throughout her pregnancy and was screened for chlamydia, gonorrhea, hepatitis B, HIV, syphilis, and group B streptococcus. These tests were negative. The infant was hospitalized in a level III NICU at the University of Chicago Comer Children’s Hospital. The Institutional Review Board of the University of Chicago approved the study and the infant’s parents provided written informed consent. All methods were performed in accordance with the relevant guidelines and regulations.

Stool, saliva, and skin swabs were collected on days of life (DOL) 8, 10, 12, 15, 18 and 21^13^. Before and during this time, the infant was treated with intravenous antibiotics, specifically, ampicillin (DOL 1–7), gentamicin (DOL 1–7 and 13–19), vancomycin (DOL 13–15) and cefotaxime (DOL 14–19). Antibiotics started on DOL 13 were given as empiric coverage for suspected sepsis; cultures of blood, urine, and cerebrospinal fluid were ultimately negative. Respiratory specimens were not tested. The infant was intubated and mechanically ventilated throughout the study period. As part of a previously published study, a portion of each sample was processed for bacterial community characterization via the sequencing of amplified 16S rRNA genes^13^. The remainders were archived at −80 °C.

*Ca*. M. girerdii-like 16S rRNA gene sequences were particularly abundant in the infant’s saliva on DOL 15 (26% of amplified reads), 18 (47%) and 21 (14%)^13^. They were not detected in the infant’s stool or on his skin, or in five other infants in the cohort^13^. We reasoned that because the novel mycoplasma had been a dominant member of a low-diversity bacterial community, namely, the oral microbiota of an antibiotic-treated premature infant, its population genome could be assembled *de novo* from metagenomic read data. We recovered relatively little genomic DNA from the infant’s saliva on DOL 15, 18 and 21; therefore, prior to sequencing, we pooled the three days (see Supplementary Methods and Table S1 for details). Paired-end, 2 × 150 bp, Illumina HiSeq sequencing yielded 19.41 million raw read-pairs.

### Assembly, preliminary annotation, and bin previews

After trimming and filtering the raw reads, 5.11 million high quality read-pairs (1.44 Gbp of data) were retained and advanced to *de novo* assembly, which, along with read mapping, gene prediction and preliminary annotation, was performed as described in the Supplementary Methods. Assembly using IDBA-UD produced 1,688 scaffolds ≥1 kbp in length, which together summed to 11.88 Mbp. An overall alignment rate of 70.3% was achieved when the assembly-input reads were mapped back to these scaffolds (improving modestly to 77.7% for all scaffolds ≥200 bp). Prior to formal binning, we found it useful to obtain an estimate of the number, types, and relative abundances of bins (genomes) present in the dataset. We did this by creating an inventory of genes encoding ribosomal RNAs (Supplementary Table S2 and Data S1) and ribosomal proteins (Supplementary Fig. S1a).

### Creating and assessing bins

Genome bins that were initially delineated using a tetranucleotide frequency-based emergent self-organizing map (ESOM) (Supplementary Fig. S2) were manually curated (i.e., validated and honed) using other genomic signatures (see Supplementary Methods for details). All scaffolds ≥1 kbp in length were either binned (n = 1413; summing to 11.45 Mbp) or removed (n = 275 residual phiX or human scaffolds). Approximately 1.15 Gbp mapped back to the binned scaffolds. This estimate included both paired and unpaired reads, but only paired reads were used in the assembly. Bins were annotated in further detail (Supplementary Table S3) and assessed for coverage, abundance, completeness, and other characteristics as described in the Supplementary Methods. Replication indices were calculated using iRep software and the thresholds recommended by the authors^18^.

### Finishing and characterizing the Mycoplasma genome

Minor local scaffolding errors were detected and corrected as described in the Supplementary Methods. The 618,983-bp *Ca*. M. girerdii str. VCU-M1 genome was used to guide initial hypotheses about the order and orientation of our scaffolds. Subsequently, we were able to close (*in silico*) the 629,409-bp genome of the infant oral strain (str. UC-B3; see Supplementary Methods for details). The finished sequence (*sensu* Sharon and Banfield)^19^ was confirmed via careful read mapping and error checking; indeed, after depleting our reads of those mapping to str. UC-B3, none remained that mapped to str. VCU-M1. The genomes were aligned (Supplementary Fig. S3) and an average nucleotide identity (ANI) was calculated; for str. UC-B3, we also generated a set of gene predictions and annotations (Supplementary Table S4), inferred its metabolic functional potential (Supplementary Table S4), and compared its genes involved in energy metabolism to those of other Mollicutes (Supplementary Table S5) as described in the Supplementary Methods.

### Analyzing length variation in DNA tandem repeats

We generated a list of candidate length-variable DNA tandem repeats, most of which were dinucleotide tandem repeats (DTRs) (Supplementary Table S6), for the finished genome of *Ca*. M. girerdii str. UC-B3. For each candidate, we quantified the types and frequencies of length variants present in the population using a read alignment and counting procedure described in the Supplementary Methods. Each length variant’s potential consequence for phase variation was inferred by translating the affiliated open reading frame (ORF) and documenting premature stop codons downstream of the repeat. Where possible, we identified the corresponding homologous repeat in str. VCU-M1 and recorded its length (Supplementary Table S7).

### Data availability

Datasets on which the conclusions of this manuscript rely have been deposited under NCBI BioProject PRJNA362245, BioSample SAMN06236793, Sequence Read Archive (SRA) accession code SRR5251628 (these are the raw reads), and Genomes accession code CP020122 (this is the complete genome sequence of Ca. M. girerdii str. UC-B3).

## Results and Discussion

### Gene inventories reveal *Trichomonas* and ample coverage of *Mycoplasma*

Inventories of genes encoding rRNAs and ribosomal proteins suggested that the premature infant’s oral metagenome comprised the genomes of as few as six genera: *Pseudomonas*, *Mycoplasma*, *Streptococcus*, *Enterobacter*, *Staphylococcus* and *Trichomonas*. The taxonomic identities and relative abundances of bacteria were similar to those observed in our earlier survey based on amplified 16S rRNA genes^13^ (Supplementary Tables S1, S2 and Fig. S1a). In addition to bacterial genomic DNA, the infant also harbored genomic DNA likely belonging to *T*. *vaginalis*, the causative agent of human trichomoniasis and a microbial eukaryote ignored by bacterial broad range 16S rRNA gene PCRs. Vertical transmission of *T*. *vaginalis* has been documented, but is considered uncommon^20–22^. As expected, the depth of sequencing achieved for the targeted *Mycoplasma*, estimated at ~80-fold average coverage (Supplementary Fig. S1a), was sufficient for genome reconstruction.

### Binning recovers three essentially complete and three partial draft genomes

As predicted from our gene inventories, binning resulted in six genome bins (Supplementary Fig. S2). Table 1 describes them in detail and Supplementary Table S3 provides basic annotations. Bins 1–3, including the target *Mycoplasma* sp. ‘Mnola’ (bin 2), were most abundant; these draft genomes were essentially complete (*sensu* Sharon and Banfield)^19^ as indicated by their size and other estimates of completeness (Table 1). Bins 4–6 were incomplete, yet offer important clues about the mycoplasma’s microbial community context, e.g., bin 6 contains 149 scaffolds assigned to *Trichomonas* (Table 1).

**Table 1:** Genomes reconstructed from the oral metagenome of a 3-week-old premature infant.

	Binned						Closed	
Genome	1	2	3	4	5	6	2	CP007711^b^
Organism	*Pseudomonas aeruginosa*	*Mycoplasma sp*. ‘Mnola'	*Streptococcus parasanguinis*	*Enterobacter cloacae*	*Staphylococcus epidermidis*	*Trichomonas vaginalis*	*Ca*. M. girerdii str. UC-B3	*Ca*. M. girerdii str. VCU-M1
Fold coverage	151.8	79.7	17.2	4.7	5.5	~0.2^a^	76.1	67–95^16^
Scaffolds ≥1 kb	71	23	95	1,013	62	149	1	1
Size (bp)	6,722,245	613,365	2,204,765	1,550,266	92,937	268,614	629,409	618,983
N50	212,032	71,441	48,153	1,504	1,401	1,817	n/a	n/a
L50	9	3	16	370	22	48	n/a	n/a
G + C (%)	66.1	28.6	41.5	55.1	32.6	31.4	28.6	28.6
Genetic code	11	4	11	11	11	1	4	4
CheckM completeness (% of marker sets)	99.68	98.08	100.00	36.95	0.00	n/d	98.08	98.08
Relative abundance (% of genomes)	58.57	30.77	6.64	1.81	2.12	0.08	n/d	n/a
Absolute abundance (% of bases)	66.58	3.19	2.48	0.48	0.03	2.31	n/d	n/a
Index of replication (iRep)	1.72	1.42	1.35	n/d	n/d	n/d	1.42	n/a
Domain; Phylum	Bacteria; Proteobacteria	Bacteria; (Tenericutes)	Bacteria; Firmicutes	Bacteria; Proteobacteria	Bacteria; Firmicutes	Eukaryota; Metamonada	Bacteria; (Tenericutes)	Bacteria; (Tenericutes)
Genes	6,512	629	2,182	2,364	123	n/d	609	611
rRNAs (*rrn* copies predicted)	5S, 16S, 23S (4)	5S, 16S, 23S (1)	5S, 16S, 23S (4)	5S, 16S, 23S (~7–8)	5S, 16S, 23S (~4–6)	5.8S, 18S, 28S (~250)^27^	5S, 16S, 23S (1)	5S, 16S, 23S (1)
tRNAs (amino acids decoded)	58 (21)	31 (20)	31 (16)	14 (10)	none detected	none detected	31 (20)	31 (20)
Other ncRNAs	n/d	RNAse P RNA	n/d	n/d	n/d	n/d	RNAse P RNA	n/d
ORFs	6,450	595	2,148	2,347	120	n/d	574	577
Frameshifts	n/d	n/d	n/d	n/d	n/d	n/d	10	4
Pseudogenes	n/d	n/d	n/d	n/d	n/d	n/d	1	1
CRISPR-Cas	none detected	none detected	none detected	present	n/d	n/a	none detected	none detected^c^

^a^Estimated using reference-based mapping; ^b^Accession number of the vaginal strain, provided here for reference; ^c^Our own finding, which differs from that of Fettweis *et al*.^16^ as described in the Supplementary Methods; n/a, not applicable; n/d, not determined; (Tenericutes) branches within the phylum Firmicutes^7^.

Owing in part, we surmised, to recent antibiotic exposure, the microbial diversity of the infant’s oral cavity was extremely low. Given the paucity of infant oral metagenomic datasets available for comparison, it is difficult to say whether this level of diversity is typical of the upper respiratory tracts of similarly treated, premature infants.

### *T*. *vaginalis* identification, coverage, and inferred route of transmission

This is the first report of a non-vaginal microbiome in which *Trichomonas* and *Mycoplasma* sp. ‘Mnola’-like sequences co-occur. We considered that (1) *T*. *vaginalis* is rarely found in neonates^23^; (2) other *Trichomonas* spp. (e.g., *T*. *tenax*) preferentially or opportunistically colonize the human oral and/or respiratory mucosa; (3) a sole species, *T*. *vaginalis*, overwhelmingly dominates the database of *Trichomonas* genomic sequence; and (4) no prior study has relied on metagenomic data to characterize *Trichomonas* spp. *in situ*. Therefore, we carefully evaluated our species-level assignment, observed patterns of assembly and coverage, and our assumptions about the mode of transmission.

Alignment of our *Trichomonas*-derived internal transcribed spacer 1 (ITS1)−5.8S rRNA-ITS2 sequence against those of other *Trichomonas* spp.^24^, followed by phylogenetic inference, indicated that *T*. *vaginalis* was the closest species. Furthermore, our *Trichomonas* 18S rRNA gene sequence was 100% identical over 1,508 positions to that of a vaginally-derived *T*. *vaginalis* isolate^25^. We concluded that ‘*T*. *vaginalis*’ was indeed the best possible species-level assignment.

At > 176 Mbp, the draft genome of *T*. *vaginalis* is >26× larger than the largest bacterial genome in our dataset. To provide landmarks without overwhelming the map, we only included the five largest *T*. *vaginalis* genome fragments in our ESOM (Supplementary Fig. S2). Using this approach, our *T*. *vaginalis*-derived scaffolds were readily binned. However, the resulting scaffolds were small in size (maximum of ~6.5 kbp), inconsistent in coverage (range ~2–200×), and summed to only 0.15% of the length of the reference genome. We sought to improve our estimate of overall coverage of *T*. *vaginalis*, which we accomplished using reference-based mapping.

We estimated an overall *T*. *vaginalis* genome coverage of ~0.2×, based on read-mapping across the entire *T*. *vaginalis* genome or on mapping to a set of *T*. *vaginalis*-specific single-copy genes (n = 16)^26^. To validate this estimate, we divided the coverage of our most highly represented (207×) *T*. *vaginalis* scaffold, encoding the 1.3 kbp Mariner transposase (repeat R8)^27^ (Supplementary Table S3), by the overall coverage (0.2×), which yielded a predicted copy number of 1035. This is close to the reported copy number of 982^27^. Repetitive sequences compose over 65% of the *T*. *vaginalis* genome, and have been shown to be highly homogenous^27^. We infer that our assembly was biased in favor of these high-copy regions. Using our reference-based estimate of *T*. *vaginalis* coverage, we propose that for every *T*. *vaginalis* genome in the infant’s pooled saliva sample (see Methods), there were roughly 385 *Mycoplasma* sp. ‘Mnola’ genomes.

*T*. *vaginalis* is associated with adverse pregnancy outcomes including preterm delivery and the delivery of a low-birthweight infant, and may increase the risk of PPROM^28, 29^. Rates of infection vary among populations, but an estimated 50% of women with *T*. *vaginalis* are asymptomatic^30–32^. We strongly suspect that the infant acquired *T*. *vaginalis* (and the uncultivated mycoplasma) at delivery via passage through the birth canal, even though to our knowledge, the mother had not been screened or treated for *T*. *vaginalis* and was presumably asymptomatic or had symptoms that did not prompt reporting, screening or treatment. We can only speculate as to whether the presence of one or both organisms played a causal role in PPROM and preterm delivery in this case.

### Evidence for *in vivo* growth of *Ca*. M. girerdii strain UC-B3

We closed the 629,409-bp genome of the infant oral *Mycoplasma* sp. ‘Mnola’ and aligned it to the closest reference, the 618,983-bp genome of *Ca*. M. girerdii str. VCU-M1 (Table 1; alignment dotplot shown in Supplementary Fig. S3). The alignment covered 96.67% and 98.18% of bases, respectively, and the average nucleotide identity (ANI) was 99.44%. The Mash distance, 0.0052, corresponded well with the ANI. Given the high degree of alignment coverage and overall nucleotide sequence similarity, we concluded that the genomes were distinct at the level of strains^33^. We applied the name *Ca*. M. girerdii str. UC-B3 (University of Chicago baby #3) to the infant oral strain. The genome-wide alignment revealed a large-scale inversion spanning str. UC-B3 positions 96–244 kbp, several smaller insertions and deletions, and numerous repetitive elements (Supplementary Fig. S3).

**Figure 1:**
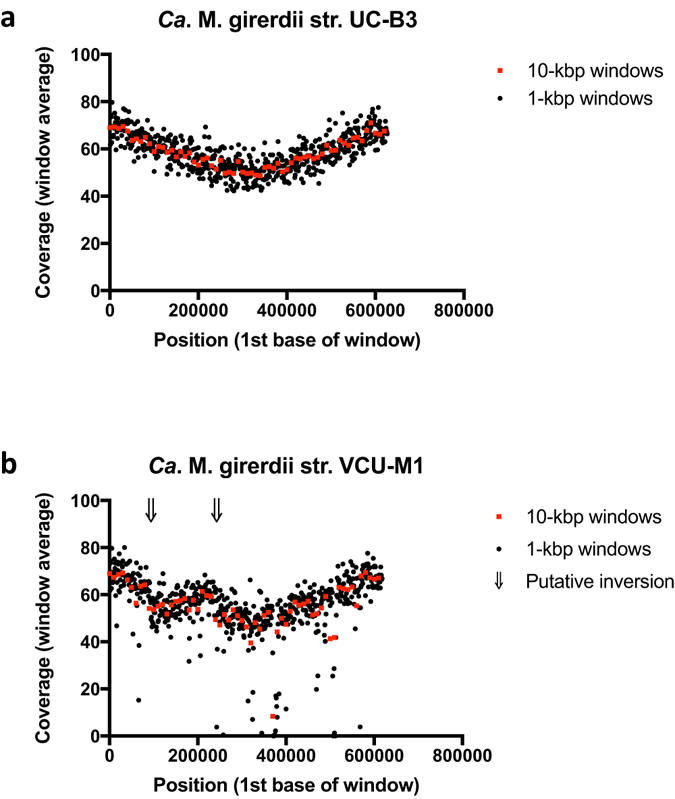
Pattern of sequencing read coverage suggests *in vivo* growth of *Ca*. M. girerdii strain UC-B3 and large-scale genome inversion with respect to strain VCU-M1. For each window, the average number of reads aligned to each base is plotted against the genome position corresponding to the first base. Windows are non-overlapping and sized at 10 kbp (red squares) or 1 kbp (black circles). For both panels, the aligned reads are those generated for the present study (i.e., the infant oral metagenomic reads). As a proxy for the origin of replication, the first base of each genome was set to the first base of *dnaA*. (**a**) Read alignment to the strain UC-B3 genome (this study) reveals a peak-to-trough ratio of ~1.4 (iRep = 1.42), suggesting active replication forks at the time of sampling. (**b**) Read alignment to the strain VCU-M1 genome^16^ confirms a large-scale inversion initially revealed by the whole genome alignment (Supplementary Fig. S3). Arrows indicate putative inversion break points.

Using the closed genome sequence, we sought evidence in support of *in vivo* growth of *Ca*. M. girerdii str. UC-B3 by examining the pattern of read coverage in relation to the origin of replication^34^. The origin was designated as described in the Supplementary Methods. Coverage decreased smoothly from origin to terminus, with coverage near the origin exceeding that near the terminus by ~1.4 fold (iRep = 1.42) (Fig. 1a). Excess copy number near the origin (i.e., a coverage ratio >1) arises from active replication forks within cells. This ratio has been shown to be directly proportional to growth rate^34^. Our data suggest that *Ca*. M. girerdii str. UC-B3 was replicating within the oral cavity of the 3-week-old premature infant. Because samples from days 15, 18 and 21 were pooled (see Methods), we cannot determine on which day(s) this occurred.

Genome closure is not required for the calculation of iRep values^18^. Therefore, we can infer from our draft genomes that *Pseudomonas aeruginosa* and *Streptococcus parasanguinis* were also replicating within the oral cavity of the infant (Table 1). By comparing iRep values, we see that *Ca*. M. girerdii str. UC-B3 may have been replicating at a faster rate than *S*. *parasanguinis*, an indigenous oral bacterium (iRep = 1.35). These results suggest that *Ca*. M. girerdii was not merely a transient contaminant in this habitat (e.g., passive material carried over from delivery). In consideration of str. UC-B3’s growth rate, we noted that in experiments carried out by Korem *et al*.^34^, a peak-to-trough ratio of 1.4 corresponded to a generation time of 1–2 hr in aerobic-, chemostat-grown *Escherichia coli*.

Finally, coverage patterns can highlight genomic rearrangements^35^. The large-scale inversion revealed by our genome-wide alignment of str. UC-B3 to str. VCU-M1 (Supplementary Fig. S3) was also apparent as an inversion in the slope of coverage when we mapped our reads to the genome of str. VCU-M1 (Fig. 1b). This inversion is not likely to be the result of misassembly. The UC-B3 assembly is supported by paired-end reads and the consistent slope in coverage, and the VCU-M1 assembly is supported by PCR products that were amplified and sequenced across the region^16^.

### *Ca*. M. girerdii encodes PFOR and is predicted to be sensitive to metronidazole

*Ca*. M. girerdii strains UC-B3 and VCU-M1 are predicted to be metabolically alike (see the Supplementary Results and Discussion for an overview of *Ca*. M. girerdii’s metabolic potential). Their predicted metabolism appears to represent a variation on the theme of mycoplasma metabolic streamlining^6^ that is particularly novel at the pyruvate locus.

*Ca*. M. girerdii is unusual among glycolytic (sugar-fermenting) mycoplasmas in lacking pyruvate dehydrogenase (PDH), the enzyme responsible for oxidatively decarboxylating pyruvate to acetyl-CoA. Instead of using PDH, *Ca*. M. girerdii likely relies on pyruvate formate-lyase (PFL) and/or pyruvate-ferredoxin oxidoreductase (PFOR), both of which were present in the two genomes (for str. UC-B3, see Supplementary Table S4). These enzymes are characteristic of anaerobically growing bacteria^36^ and have not been detected in other mycoplasmas (Supplementary Table S5).

PFL produces acetyl-CoA and formate from pyruvate and coenzyme-A. PFL requires a ‘pyruvate formate-lyase activase’ and co-occurs with a replacement part, an ‘autonomous glycyl radical cofactor’, both of which were present in the two genomes. The radical cofactor is thought to quickly restore activity in stress-damaged PFL. Among Mollicutes, PFL has been detected in a few *Spiroplasma* spp. and in *Acholeplasma* spp. (Supplementary Table S5), the latter occupying the taxonomically ambiguous base of the class Mollicutes, which branches *within* the phylum Firmicutes^7, 37^. PFOR produces acetyl-CoA, CO_2_, H^+^ and reduced ferredoxin from pyruvate, coenzyme-A and oxidized ferredoxin. PFOR is typically coupled to a hydrogenase that is responsible for re-oxidizing the reduced ferredoxin^36^. We identified genes encoding ferredoxins in the *Ca*. M. girerdii genomes, but were unable to identify genes encoding hydrogenases. Thus, alternatively, PFOR may operate in reverse and couple with PFL to reduce CO_2_ to formate, which may then feed into reductive one-carbon metabolism^38^. PFOR is found in *Acholeplasma brassicae*, *Acholeplasma morum*, *Ca*. Izimaplasma spp., and on several contigs assembled from human gut metagenomes (so-called ‘CAG’ entities) (Supplementary Table S5)—all of which are predicted to be ‘basal’ Mollicutes in the manner noted above^37, 39^. *Ca*. M. girerdii’s PFOR is highly novel and its presence is not likely to be explained by recent lateral transfer. Its 1192-aa sequence is in the range of 53–55% identical to PFORs from a diverse set of Firmicutes species.

Finally, if PFOR were active, we would expect *Ca*. M. girerdii to be sensitive to metronidazole, the antibiotic commonly used to treat infections caused by anaerobes including *T*. *vaginalis*, but not used in this infant. PFOR reduces metronidazole to its active form. Because *Ca*. M. girerdii str. UC-B3 persisted and possibly bloomed in the face of antibiotic exposure, we also looked for putative resistance mechanisms (see Supplementary Methods). On balance, it seemed likely that intrinsic resistance mechanisms (e.g., lack of a cell wall), which could be inferred for all mycoplasmas, played a larger role than acquired ones in str. UC-B3 (see Supplementary Results and Discussion for details).

### Genomic features distinguishing *Ca*. M. girerdii str. UC-B3 from str. VCU-M1

The *Ca*. M. girerdii genomes are AT-rich, small, and use genetic code four (UGA codes for Trp instead of Stop) (Table 1). These traits are common among the *Mycoplasmataceae*. The genomes also reflect the phylogenetic novelty first noted in their 16S rRNA genes (see Introduction)^13, 15^. The genomes of the most closely related organisms, e.g., *M*. *iowae* and *M*. *penetrans* (of the *M*. *pneumoniae* group *sensu* Davis *et al*.^37^) (Supplementary Fig. S4), are not broadly alignable to those of *Ca*. M. girerdii at the level of nucleotide sequences.

**Figure 2:**
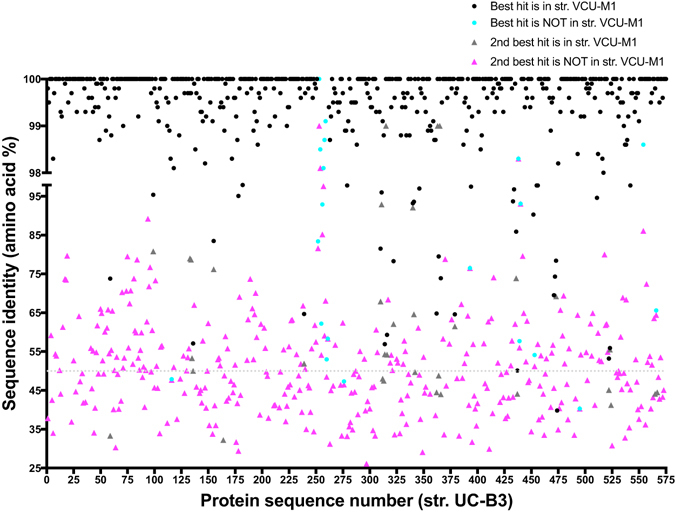
Phylogenetically distinct from other Mollicutes, *Ca*. M. girerdii strains are distinguished from each other by a small set of variable features. Displayed are the highest identity database hits for *Ca*. M. girerdii strain UC-B3 proteins. Sequences (n = 574) were queried against the UniRef100 database (downloaded December 2015) using USEARCH in local alignment mode with the settings described in the Supplementary Methods. Under these search conditions, 8 sequences had no hit (not plotted), 176 had only 1 hit (plotted as ‘best’), and 390 had at least 2 hits (plotted as ‘best’ and ‘2^nd^ best’ by identity). Also indicated is whether or not the hit was from closely related strain VCU-M1. USEARCH is most effective at protein identities ≥50% (dotted line). On the x-axis, sequences are numbered in order of appearance in the UC-B3 genome.

Only 63% of str. UC-B3 proteins had a valid hit in a non-VCU-M1 genome; among these hits, the median level of sequence identity was 50% (Fig. 2). Most non-VCU-M1 hits (~70%) were to other Mollicutes, with the rest mainly to Firmicutes. These findings confirm the designation of a new species made by Fettweis *et al*.^16^, and are notable because uncultivated organisms with a high degree of phylogenetic novelty are rarely encountered in human body habitats (although see refs 40, 41).

**Figure 3:**
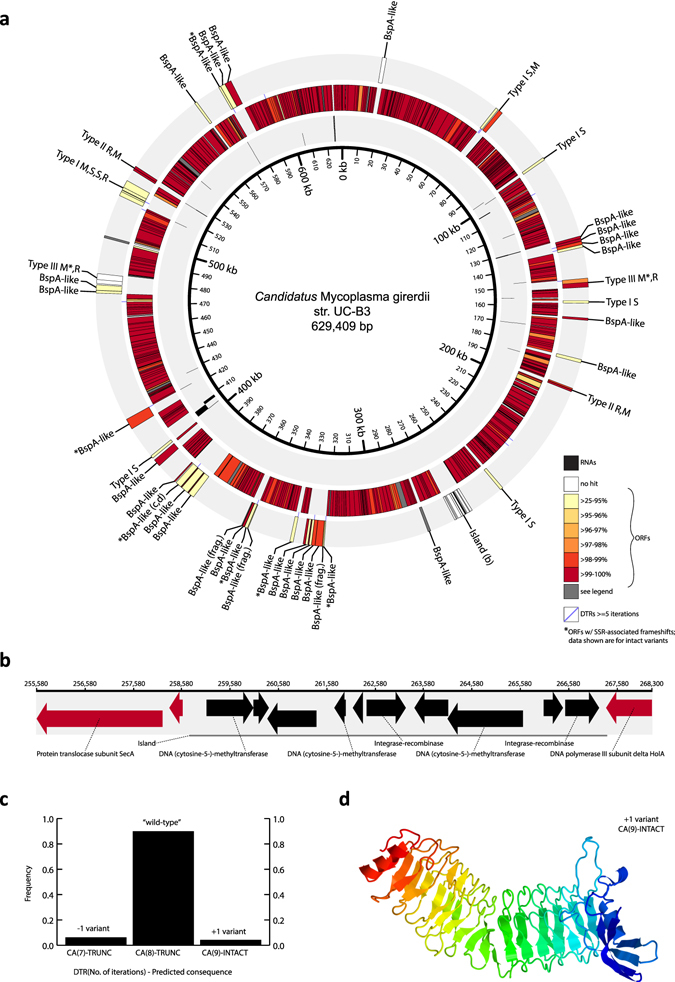
Genomic features distinguishing *Ca*. M. girerdii strain UC-B3 from strain VCU-M1 include those with evidence of phase variation within population UC-B3. (**a**) Circular genome map of *Ca*. M. girerdii strain UC-B3. Inner track shows RNA genes (black; staggered for clarity). Center and outer tracks show open reading frames (ORFs; color graded on percent amino acid identity to reciprocal best hit in VCU-M1). ORFs in gray have a close nucleotide hit in strain VCU-M1 but were not predicted there by the authors. Purple lines, located between center and outer tracks, represent dinucleotide tandem repeats (DTRs) of five or more iterations (Supplementary Table S6). ORFs from variable classes were manually placed in the outer track. Those with asterisks display evidence of phase variation (Supplementary Table S7); those without labels (n = 5) encode fructose-bisphosphate aldolase; and those composing restriction modification systems are labeled clockwise as follows: R, restriction endonuclease; M, modification methylase; S, specificity subunit. The subjects of panels b–d are labeled in bold. SSR, simple sequence repeat. (**b**) Linear map detailing genomic island in UC-B3, which is absent from VCU-M1. ORFs drawn as arrows; those without labels encode hypothetical proteins. (**c**) Evidence of phase variation for selected feature no. 11 in Supplementary Table S7. Plotted are length variant frequencies for DTR ‘CA’ associated with BspA-like protein labeled in bold in panel a. Homologous ORF in VCU-M1 contains CA(10) (Supplementary Table S7). TRUNC, frameshift-induced premature stop codon resulting in truncated BspA-like protein; INTACT, likely produces intact BspA-like protein shown in panel (d). The intact protein was modeled against the highest scoring template (pneumococcal vaccine antigen PcpA) using Phyre2^55^. Left/red, N-terminus; right/blue, C-terminus.

Most str. UC-B3 protein-coding genes (n = 529 of 574) had an easily-identified ortholog in str. VCU-M1, defined here as a syntenic, high-identity (≥97% nucleotide identity), reciprocal best hit (see Supplementary Results and Discussion for details). We defined those without easily-identified orthologs (n = 45) as UC-B3′s ‘variable set’ (Supplementary Table S4). Most ‘variable set’ genes (n = 40) could be grouped into one of four distinct classes, representing a first glimpse into the major themes of strain-level differentiation in *Ca*. M. girerdii. These four classes, and the features composing them (Fig. 3a,b), are described in detail in the Supplementary Results and Discussion.

The ‘variable set’ included genes encoding a diverse array of BspA-like surface proteins (named for *B*. *forsythus* surface protein A), of which str. UC-B3 contains at least 28 distinct copies, as well as those encoding restriction-modification (R-M) systems (str. UC-B3 contains 18 genes involved in R-M systems) (Fig. 3a). Notably, *T*. *vaginalis* also encodes a diverse array of BspA-like surface proteins^27^. BspA was identified in *Tannerella* (formerly *Bacteroides*) *forsythus*, contains a structural motif known as a *Treponema pallidum* leucine-rich repeat (TpLRR), and is thought to be involved in host cell attachment and invasion, co-aggregation, and the stimulation of immune responses^42–44^. Genomic regions exclusive to str. UC-B3, including an 8.6-kbp genomic island carrying cytosine methyltransferases (Fig. 3b), tend to reflect recent lateral exchange with *Mycoplasma hominis* (see Supplementary Results and Discussion). Given *Ca*. M. girerdii’s highly reduced genome size and metabolic repertoire, expansion and elaboration within these classes of genes almost certainly highlights ecological strategies that are important to the organism, which may include attachment, immune evasion, and defense against foreign DNA.

### Length variation in DNA tandem repeats within population UC-B3

Natural populations are rarely isogenic. During the sampling period, str. UC-B3 was likely replicating *in vivo* (Fig. 1a). Therefore we investigated within-population variation in str. UC-B3, as a window onto recent diversification. We began with routine variant calling—mapping reads to the UC-B3 genome and identifying polymorphic sites (mainly SNPs and short indels). We found relatively few such sites: ~50, depending on the stringency of read mapping and variant calling. Salient among them were indels of dinucleotides at dinucleotide tandem repeats (DTRs). We also noticed that many of UC-B3’s polymorphic DTRs appeared within or near genes belonging to classes likely to exhibit phase variation (e.g., BspA-like surface proteins; HsdS specificity proteins of type I R-M systems). We therefore undertook a systematic analysis in which we (1) located all simple sequence repeats in the str. UC-B3 genome, (2) identified those exhibiting length variation, (3) determined the frequencies of length variants, and (4) predicted possible consequences for associated gene expression (i.e., phase variation) as described in the Supplementary Methods.

In population UC-B3, all observed indels of iterations of simple sequence repeats appeared in repeat tracts ≥5 iterations in length, most of which were DTRs. The UC-B3 genome contains 20 DTRs ≥5 and ranging up to 12 iterations in length (Fig. 3a and Supplementary Table S6). Eight different motifs are represented (all but ‘tg’, ‘gt’, ‘gc’ and ‘cg’) (Supplementary Table S6). Of the 20 UC-B3 DTRs, 19 have a clear homolog in str. VCU-M1, and 12 of these differ in the number of iterations (Supplementary Table S7). Of the 20 UC-B3 DTRs, 13 exhibited differences in the number of iterations *within* the UC-B3 population (Supplementary Table S7). In all cases, as expected, the DTR in the UC-B3 genome (i.e., the wild-type) was most frequent. We also noted that all length variants featured indels of one or more dinucleotide iterations, and not of odd numbers of nucleotides, or of differing bases, as might occur by chance or error (see Supplementary Results and Discussion). In addition to the DTRs, we identified two, length-variable homopolymeric tracts (Supplementary Table S7).

The frequencies of length variants at repeat sites (average 0.082; range 0.017–0.446) (Supplementary Table S7) far exceeded those of dinucleotide indels at non-repeat sites (see Supplementary Methods) and those expected for indel sequencing errors^45^ (see Supplementary Results and Discussion). Affected genes included eight BspA-like surface proteins; non-solitary HsdS proteins associated with the type I R-M loci; and methyltransferases associated with type III R-M loci (Fig. 3a and Supplementary Table S7). With respect to phase variation, for a number of affected genes, the wild-type is predicted to be ‘off’ (i.e., not expressed or severely truncated) owing to frameshift mutations that induce premature stop codons (i.e., early termination), while one or more low-frequency variants are predicted to be ‘on’ (Supplementary Table S7). An example of this is shown in Fig. 3, in which only the +1 DTR variant produces an intact BspA-like protein (Fig. 3c,d). For the two HsdS loci, each containing two length-variable DTRs, the consequences for phase variation are more difficult to predict, as outlined in Supplementary Table S7. Both type III R-M methyltransferases (see Supplementary Results and Discussion) are phase ‘off’ in wild-type UC-B3, with the *M*. *hominis*-like *mod* controlled by a homopolymer tract G(8), rather than by a DTR (Supplementary Table S7).

Phase variation randomly and reversibly generates phenotypic diversity within clonal populations via heritable genetic or epigenetic means, typically manifesting in ‘on/off’ patterns of gene expression (i.e., switching)^46^. This allows small populations of bacteria to rapidly explore new niches, or to evade the host immune system when colonizing a new host individual. Phase variation is well-documented among the types of genes in which it was observed here^47, 48^. Phase variation of components of R-M systems has been proposed to either facilitate the acquisition of potentially beneficial foreign DNA^49, 50^ or to regulate expression of suites of genes^51^. Although sequence read data have been used to assess the frequency of tract length variants in a laboratory-grown population of *Helicobacter canadensis* ^52^, to our knowledge, ours is the first study to do so using metagenomic read data and a *de novo* assembled genome from a natural population. Although our data are short reads and therefore the variants remain unlinked, we suggest that the *Ca*. M. girerdii str. UC-B3 population comprised multiple subpopulations expressing unique arrays of e.g., BspA-like surface proteins.

## Conclusions

Vast, unexplored regions of the tree of life have been uncovered by sequence-based surveys of the microbial world. This phylogenetic *terra incognita* encompasses countless diverse lineages, many of which have been repeatedly detected in the environment but otherwise remain mysterious. This so-called ‘microbial dark matter’ is encountered most places microbes naturally reside, including the human body. The current study stands as an example of how genome-resolved metagenomics provide a powerful and reproducible means of shedding light on the ecology and evolution of such lineages—here, an emerging uncultivated mycoplasma, *Ca*. M. girerdii, associated with trichomoniasis in women^15, 16^. By uncovering all forms of variation, these approaches offer unparalleled access to the dynamics of the human microbiome.

We found that the oral cavity of a vaginally-delivered, three-week-old premature infant harbored both *Ca*. M. girerdii and *T*. *vaginalis*. Vertical transmission of these organisms from an asymptomatic mother is surmised; however, a weakness of our study is its lack of clinical data from the mother. For example, we do not have direct evidence that she carried *T*. *vaginalis*. Nevertheless, chance findings such as these remind us of the need for careful screening prior to the deliberate transfer of maternal vaginal microbes to Cesarean-delivered neonates^53^. Because our work suggests that the association of *Ca*. M. girerdii with *T*. *vaginalis* could extend to the premature infant upper respiratory tract, follow-up studies would be warranted in which the microbiomes of pregnant women and their newborns were monitored over time in patient populations at high risk of trichomoniasis and preterm birth, using methods capable of detecting *T*. *vaginalis*.

We found that the assembled genome of the infant oral strain, *Ca*. M. girerdii str. UC-B3, exhibited excess copy number near the origin of replication. From this we inferred a positive growth rate—possibly a relatively fast growth rate, especially for a mycoplasma—at some point during the infant’s third week of life. We also found that str. UC-B3 demonstrated high-frequency variation in the lengths of DNA tandem repeats, likely capable of inducing the ‘on/off’ switching of gene expression (i.e., phase variation). Given the types of genes affected, we infer that str. UC-B3 exhibited cell-to-cell variation in its expressed repertoires of BspA-like surface proteins and restriction-modification systems. Well-known among mycoplasmas and other, mainly pathogenic bacteria, phase variation is a strategy that facilitates colonization, evasion of host defenses, and protection of cells from phage and foreign DNA^2, 50, 51, 54^. To date, metagenomic approaches have not been used to study phase variation; however, our results suggest they are well-poised to contribute to our understanding of these dynamics *in vivo*, e.g., using time series.

Finally, we and others^16^ have found that *Ca*. M. girerdii uses metabolic circuitry that is distinct from all other mycoplasmas, and from most true Mollicutes, possibly reflecting a unique history of genome reduction in the same niche as, or in close association with the anaerobic, hydrogenosome-bearing eukaryote *T*. *vaginalis*. For example, *Ca*. M. girerdii and *T*. *vaginalis* may share the capacity for anaerobic pyruvate metabolism via pyruvate-ferredoxin oxidoreductase (PFOR). A question arising from this finding is whether the presence of *Ca*. M. girerdii influences the effectiveness of metronidazole, a drug activated by PFOR, in treating trichomoniasis. Beyond this, the nature of their association, including its clinical relevance, as well as its distribution across animal host species, remains unknown. Certainly an important unresolved question is whether *Ca*. M. girerdii modulates the pathogenicity of, or host response to *T*. *vaginalis*.

## Electronic supplementary material

Supplementary Text and Figures S1-S4

Supplementary Tables S1-S7

Supplementary Data S1
