# Supplementary material for: Candidatus Mycoplasma girerdii replicates, diversifies, and co-occurs with Trichomonas vaginalis in the oral cavity of a premature infant
Source: Sci Rep. 2017 Jun 19;7:3764. doi: 10.1038/s41598-017-03821-7 (PMC5476646; doi:10.1038/s41598-017-03821-7)
Supplement: Supplementary file 1 — Supplementary Text and Figures S1-S4 [file 41598_2017_3821_MOESM1_ESM.pdf]

Supplementary Information

Candidatus Mycoplasma girerdii replicates, diversifies, and co-occurs with *Trichomonas vaginalis* in the oral cavity of a premature infant

Elizabeth K. Costello[1], Christine L. Sun[2], Erica M. Carlisle[3], Michael J. Morowitz[4], Jillian F. Banfield[5], David A. Relman[1,2,6*]

[1]Department of Medicine, Division of Infectious Diseases, Stanford University School of Medicine, Stanford, CA 94305, USA.

[2]Department of Microbiology & Immunology, Stanford University School of Medicine, Stanford, CA 94305, USA.

[3]Department of Surgery, Division of Pediatric Surgery, University of Iowa College of Medicine, Iowa City, IA 52242, USA.

[4]Department of Surgery, Division of Pediatric Surgery, University of Pittsburgh School of Medicine, Pittsburgh, PA 15224, USA.

[5]Department of Earth & Planetary Science, University of California, Berkeley, CA 94720, USA.

[6]Veterans Affairs Palo Alto Health Care System, Palo Alto, CA 94304, USA.

[*]To whom correspondence should be addressed: relman@stanford.edu

Supplementary Methods

DNA extraction and shotgun sequencing

Genomic DNA was extracted from archived saliva using a MO BIO PowerSoil DNA Isolation kit with the following modifications: (1) before bead beating, bead tubes containing sample and solution C1 were incubated at 65°C for 10 minutes and (2) to diminish shearing, the duration of bead beating was reduced from 10 to 2 minutes. To meet the DNA mass requirement for sequencing, it was necessary to pool the selected time points (DOL 15, 18 and 21). Supplementary Table S1 shows the 16S rRNA-based taxonomic profile and amount of genomic DNA recovered from each time point. This provides a genus-level overview of the types and abundances of bacterial genomes we expected to recover.

Paired-end, 2 × 150 bp sequencing using an Illumina HiSeq2500 instrument was carried out at the Roy J. Carver Biotechnology Center at the University of Illinois at Urbana-Champaign. Prior to sequencing, the genomic DNA was fragmented to an average size of 450 bp (range of 150 to 1,000 bp) in a Covaris M220 Focused-ultasonicator. The sequencing library was constructed using a KAPA Library Preparation kit (Kapa Biosystems), multiplexed with 9 other libraries, and sequenced using a TruSeq Rapid SBS kit (v1). Fastq files were generated with Casava v1.8.2. The sequencing yield was 19.41 million raw read-pairs.

Trimming and filtering raw reads

Residual adaptors were stripped using SeqPrep (v1.1; https://github.com/jstjohn/SeqPrep). Low quality regions were trimmed using sickle software in paired-end mode with a minimum remaining length threshold of 50 bp (v1.210; https://github.com/najoshi/sickle). Reads aligning to the phiX or human reference genomes were discarded using Bowtie 2 (v2.2.1)[1]. After applying these filters, 5.11 million high quality read-pairs remained and were advanced to assembly; for these pairs, the average (SD) lengths of read 1 and 2 were 144 (16) and 137 (25) bases, respectively. Of the 74% discarded raw reads, most mapped to the human genome. High quality unpaired reads (0.45 million in total) were not used in assembly, but were used in coverage calculations.

Assembly, gene prediction and preliminary annotation

De novo assembly was carried out using IDBA-UD (Iterative De Bruijn Graph De Novo Assembler for Short Reads Sequencing Data with Highly Uneven Sequencing Depth; v1.1.1)[2]. This produced 21,375 contigs and 18,866 scaffolds ≥ 200 bp in length (the longest scaffold was 598,570 bp). Using Prodigal in metagenomic mode (v2.60)[3], we predicted open reading frames (ORFs) for all scaffolds ≥ 200 bp. This produced 34,518 ORFs encoding protein sequences ≥ 20 amino acids in length. These sequences were queried against the UniProt UniRef100 database (downloaded August 2014)[4] using USEARCH (v7.0)[5] in local alignment mode with a maximum E-value of 1e-10, minimum identity of 0.25, minimum fraction of query covered by the alignment of 0.75, maxaccepts of 8, and maxrejects of 256. UniRef100 database entries list both the protein cluster and

organismal representative; thus, our hits were tagged with taxonomic information. To estimate coverage, paired and unpaired reads (the output of sickle) were mapped to scaffolds using Bowtie 2, and the scaffold lengths and aligned read counts were parsed from the Bowtie output (SAM file). Finally, scaffold %G+C values were parsed from the Prodigal output (GBK file).

Ribosomal RNA genes were predicted on *de novo* assembled scaffolds using RNAmmer (v1.2)[6] (Supplementary Table S2). Template-guided assembly of full-length SSU rRNA genes was carried out using EMIRGE and the 'standard candidate SSU' database[7] (https://github.com/csmiller/EMIRGE; Supplementary Data S1).

Bin previews

The purpose of bin previews is to estimate the number, types, and relative abundances of bins present in the dataset prior to formal binning. Because the only available sequence representing the target mycoplasma was (at the time) its amplified 16S rRNA gene sequence, we first confirmed its presence among the rRNA genes assembed from the metagenome. As noted above, rRNA genes were obtained by assembling them directly from reads using EMIRGE and by predicting them on IDBA-UD-assembled scaffolds using RNAmmer. Next, we gathered all sequences annotated as ribosomal proteins and sorted them by type, scaffold coverage, and organismal taxonomy. Unlike for rRNA genes, most genes encoding ribosomal proteins appear in single copy; therefore, the inventory can be used to preview bin rank abundances (Supplementary Fig. S1a). We also previewed bins by plotting scaffold coverage against %G+C (Supplementary Fig. S1b).

Binning – overview and strategy

De novo assembly of metagenomic read data results in a pool of fragments (here, scaffolds) derived from a mixture of genomes. To examine each genome individually, one must parcel out (or 'bin') the fragments based on some measure of similarity. This process assumes that fragments derived from the same population genome have similar genomic signatures. Here, we assessed scaffold similarity using tetranucleotide frequencies, coverage, %G+C, genetic code, and taxonomic affiliation. When considered together, these signatures allowed us to unambiguously bin each scaffold to a genome.

Bin previews suggested the infant's oral metagenome contained as few as six microbial genome types. Therefore, we first explored a relatively coarse-grained approach to initial clustering: a plot of coverage versus %G+C for all scaffolds ≥ 10 kbp in length, which revealed three distinct clusters (Supplementary Fig. S1b), likely corresponding to the most abundant genomes (Supplementary Fig. S1a). Extending this plot (to include all scaffolds ≥ 1 kbp) uncovered additional clusters, but also blurred the boundaries among most of them (Supplementary Fig. S1b). Recognizing a need for better resolution, we ultimately prioritized an initial clustering method based on tetranucleotide frequencies.

Binning - tetranucleotide frequencies and ESOM

Scaffold fragments with similar tetranucleotide frequencies were clustered and visualized using an emergent self-organizing map (ESOM) according to the methods described in (and scripts made available by) Dick *et al.*[8] (https://github.com/EnvGen/Binning). Databionic ESOM Tools (v1.1)[9] were used to generate and visualize the ESOM and to select and export the clusters (i.e., putative bins). Reference genomes, listed in Supplementary Fig. S2, provided landmarks. This analysis was performed on all scaffolds ≥ 1 kbp in length (n = 1,688) and the fragment length ranged from 1,000 to 9,951 bp (average of 4,520 ± 1,370 bp). An initial round of ESOM revealed several clusters composed of residual phiX and human scaffold fragments (n = 272 scaffolds); these were removed from the final round depicted in Supplementary Fig. S2.

Binning - other genomic signatures

Scaffold coverage, %G+C, genetic code, and taxonomic affiliation were also considered for the purposes of binning. Scaffold coverage and %G+C were obtained as described above. We parsed the genetic code ('transl_table' field) from the Prodigal output (GBK file). (The *Mycoplasmatales* use genetic code four, in which UGA codes for tryptophan instead of 'stop'.) As noted above, taxonomic information accompanied the UniRef100-based annotations. We also tagged scaffolds with taxonomic information by querying them against a small database using BLAST and the QIIME script assign_taxonomy.py[10]. The database consisted of the reference genomes used as ESOM landmarks (Supplementary Fig. S2; except the database included the entire *Trichomonas vaginalis* genome). Scaffolds with valid hits were assigned the taxonomic string affiliated with the hit. Bin assignments were manually curated. To facilitate this process, all signatures were integrated into a common database and considered jointly.

Bin annotation

Bacterial genomes (n = 5) were annotated using RAST (Rapid Annotation using Subsystem Technology) and the default RASTtk pipeline (v2.0)[11,12]. This provided preliminary information about each bin's complement of protein-, rRNA-, and tRNA-encoding genes, as well as of CRISPR-Cas systems and other repeats. (RAST uses Prodigal to predict ORFs.) We also queried the RAST-predicted protein sequences against the UniRef100 database as described above. Bin annotations are provided in Supplementary Table S3.

RAST identified a CRISPR-Cas system in our *Enterobacter* partial genome (Supplementary Table S3 bin4). Because genome assembly may overlook these and other repetitive elements, we also performed a dataset-wide read-based search for CRISPRs using Crass software (v0.3.12)[13]. This confirmed the RAST-identified CRISPR in *Enterobacter* and did not uncover any additional valid loci.

To identify proteins potentially involved in antibiotic resistance, we used HMMER3[14] to query our predicted protein sequences against a database of antibiotic resistance-specific profile HMMs (v1.2; updated January 2015)

using the same search parameters as in Gibson *et al.*[15]. The results of this search are provided in Supplementary Table S3.

For the *Trichomonas* partial genome, we did not predict ORFs per se because Prodigal is optimaized for bacterial and archaeal genomes, and because detailed eukaryotic gene calling was beyond the scope of this study. To obtain annotation information, we performed translated nucleotide searches using BLASTX against two protein databases: the *T. vaginalis* reference genome (strain G3; NCBI accession number NZ_AAHC01000000) and the UniRef100 database (results provided in Supplementary Table S3 bin6). An initial search against the latter database uncovered nine small non-*Trichomonas* scaffolds that were either re-binned (3 *Enterobacter*, 3 *Staphylococcus*) or removed (3 human).

Bin assessment

We estimated genome coverage by dividing the number of bases aligned to the genome by the length of the genome (see next paragraph for coverage of *Trichomonas*). Genome relative abundance was calculated by dividing the genome coverage by the sum of all genome coverages[16]. Genome absolute abundance was calculated by dividing the number of bases mapped to the genome by the number of bases used in the assembly[16]. Genome completeness was assessed using CheckM software (v1.0.3)[17] and is based on the percentage lineage-specific marker gene sets present in the genome. Other features such as N50 and L50 were evaluated using scripts made available here: https://github.com/Geo-omics/scripts. Bin assessment data are provided in Table 1.

***Trichomonas* coverage**

Using BLAST and BLASTX searches, we examined our longest *Trichomonas* scaffolds (each ~4-6.5 kbp) and found they encoded features that were likely repetitive[18]. One of them, a scaffold with 124× coverage, encoded rRNA genes; indeed, *T. vaginalis* contains ~250 copies (of so-called 'rRNA units')[18]. A low level of repeat polymorphism is also reported for the *T. vaginalis* genome[18]. Considering this and the small size of our bin (0.15% the length of the reference genome), it seemed likely that our genome-wide coverage was < 1×. To estimate the overall coverage in a manner uncomplicated by copy number, we ultimately mapped our reads to the *T. vaginalis* reference genome using Bowtie 2. Dividing the number of bases mapped by the length of the reference genome, we estimated an overall coverage of ~0.2×. A similar level of coverage was found when we mapped our reads to a set of 16 *T. vaginalis*-specific single-copy genes[19].

Manual improvement of *Mycoplasma* scaffolds

The 23 scaffolds initially binned to *Mycoplasma* were checked for assembly errors by aligning to them our high-quality read-pairs under stringent conditions using Geneious (v7.1.4)[20] (http://www.geneious.com). Among five scaffolds, eight small coverage gaps were detected. Read and contig mapping indicated that these gaps were associated with minor scaffolding errors made by the assembler IDBA-UD. Using the mapping data, we were able

to manually correct these errors (as in Brown *et al.*[21]). Stringent read mapping to the corrected scaffolds revealed no further gaps in coverage, nor any areas of significantly reduced coverage.

In silico finishing of *Mycoplasma* genome

Only one of our draft genomes, that of the uncultivated *Mycoplasma* sp. 'Mnola', was subjected to finishing and detailed characterization. The others remain in 'essentially complete' or partial draft form (*sensu* Sharon and Banfield[22]).

We aligned the 23 corrected *Mycoplasma* scaffolds to the closest reference genome (*Ca.* M. girerdii strain VCU-M1[23]; accession number CP007711) using LASTZ from within Geneious. By consulting these alignments, we were able to make initial predictions about gap edges and sizes (i.e., which scaffolds to join and how far apart they might be).

Scaffolds bridged by putative gaps, represented by strings of Ns of the predicted size, were passed to GapFiller (v1.10)[24]. Using Bowtie-mapped read-pairs, GapFiller iteratively extends sequences flanking gaps until the gaps are closed. GapFiller depends not only on the accurate input of starting edges, but also of starting gap sizes. In some cases, closure was achieved only after incrementally altering the starting gap size. Although gaps were initially resolved one at a time, in the end, all gaps in the draft genome (a version of the genome in which all gaps were represented by Ns) could be closed in a single GapFiller run at m = 100 ('m' represents the minimum number of overlapping bases with the sequences flanking the gap).

The validity of the 629,409-bp finished genome was checked using stringent read mapping in Geneious, which failed to detect any gaps in or areas of aberrant coverage. Because we binned scaffolds ≥ 1 kb in size, we also checked whether shorter fragments, those not considered for binning, could be recruited to the filled gaps. When we mapped all of our IDBA-UD assembled scaffolds to the finished genome, we recruited an additional 73 short scaffolds (ranging in length from 126 – 835 bp). Among them, 19 (ranging in length from 139 – 327 bp) mapped perfectly to more than one location. Mapping all 96 scaffolds in a manner that allowed the 19 mapping more than once to do so, covered 628,674 (99.9%) of the 629,409 bases.

We also used re-assembly to validate the finished genome. *Ca*. M. girerdii reads were re-assembled using IDBA-UD (as described above) and also SPAdes (v3.5.0)[25]. SPAdes was executed in 'only-assembler' and 'careful' modes, with a coverage cutoff value of 20. All re-assembled fragments mapped to the finished genome. The SPAdes results were particularly useful: SPAdes-assembled scaffolds spanned a majority of gaps, which helped to confirm the gap-filled sequences.

Finding no clear signal in the pattern of DNA compositional asymmetry, and no obvious clusters of DnaA boxes (none were detected in strain VCU-M1 either[23]), we designated the first base of *dnaA* as the first base of the genome, assuming an origin nearby (as is often the case[26]).

Whole genome alignment and average nucleotide identity

NUCmer (MUMer v3.23)[27] was used to align the finished genome of *Ca*. M. girerdii strain UC-B3 (this study) to that of *Ca*. M. girerdii strain VCU-M1[23] (Supplementary Fig. S3) and, using the dnadiff wrapper, to calculate the average nucleotide identity (ANI). To complement these results, we also computed a Mash distance, which closely approximates ANI but is based on k-mers, using Mash software (v1.1)[28].

Gene prediction, annotation, and metabolic reconstruction

To annotate the finished genome of *Ca*. M. girerdii strain UC-B3, we employed a strategy in which we gathered and compared annotations from multiple sources. This approach was motivated by the fact that most *Ca*. M. girerdii sequences are highly dissimilar from those found in publically available databases (e.g., see Fig. 2).

We used Prodigal to predict ORFs for the finished genome. The resulting protein sequences were subjected to homology-based searches against UniRef100[4], the SEED (via RASTtk)[11], KEGG GENES[29], and NCBI nr; and to model-based signature recognition searches using InterProScan (v5)[30]. Annotations from all sources were integrated into a common database and considered jointly before selecting the best, and most consistent, final annotation (results provided in Supplementary Table S4). Out of 574 predicted proteins, we assigned a specific funtion to 424, a generic function (e.g., family or domain) to 55, and no function (i.e., hypothetical) to 95. In a few cases, close examination of the annotation data prompted manual modification of the Prodigal-predicted ORF (e.g., adjusted the start site). These instances are flagged in Supplementary Table S4. To generate the data shown in Fig. 2, we used an updated version of the UniRef100 database to which *Ca*. M. girerdii strain VCU-M1 had been added (downloaded December 2015).

The LSU and SSU rRNA genes identified by RASTtk were confirmed by aligning them to the Silva database (http://www.arb-silva.de)[31]. Transfer RNA genes were identified using tRNAscan-SE (v1.21)[32], with refinements to functional classifications made using the TFAM Webserver (v1.3)[33]. The ribonuclease P RNA gene was identified using the Ribonuclease P Database[34] and the methods of Li and Altman[35].

Metabolic pathway and other functional category assignments were made using KEGG Mapper[29], followed by manual curation (results provided in Supplementary Table S4). References consulted during manual curation included the MetaCyc database[36], IMG database[37], and White's textbook[38]. IMG was used to compare Mollicutes genomes with respect to the presence/absence of genes involved in energy metabolism (Supplementary Table S5).

Analysis of length variation in DNA tandem repeats

Simple sequence repeats (e.g., dinucleotide tandem repeats) ≥ 5 iterations in length were identified in the genome of *Ca*. M. girerdii strain UC-B3 using the IMEx webserver[39]. Among these, dinucleotide tandem repeats were particularly common (Supplementary Table S6). Geneious was used to annotate the repeats, and to perform genome-wide read mapping and variant calling. By examining each tract, we identified those for which some fraction of the mapped reads exhibited length variation (i.e., insertions/deletions of repeat iterations). We also consulted the list of variant calls made by Geneious, which revealed length variation at several other loci (an imperfect dinucleotide repeat and two homopolymer tracts) (Supplementary Table S7). Iterating through these steps, we generated a list of candidate length-variable loci.

Next, we quantified the types and frequencies of length variants at each locus. This required correct alignments. Because automated aligners often mis-align simple sequence repeats (e.g., inserting gaps into different places in identical reads), we performed the following steps: (1) extracted all read fragments completely spanning the tract (i.e., 'bookends' were required), (2) re-aligned the fragments to the tract automatically, (3) corrected the alignments manually, and (4) re-calculated the variant frequencies, counting read-pairs only once (reassuringly, for pairs in which both reads spanned the repeat, the length variants always matched). The average frequency of length variants at repeat sites was 0.082 (range 0.017 – 0.446) (Supplementary Table S7). For comparison, the deletion of any dinucleotide (e.g., one 'at', 'tt', etc) at a minimum frequency 0.01 was observed at 39 bona fide non-repeat sites (i.e., sites that did not feature repeats of the dinucleotide). Among these sites, the dinucleotide deletion frequency was on average 0.018 and maximally 0.039. Therefore, the highest (by far) deletion frequencies were observed at repeat sites; these frequencies may have been shaped by mutation (slipped-strand mispairing) +/- selection for particular variants. However, it is important to point out that some of the frequencies observed at these sites were within the range observed at non-repeat sites.

At all loci, the assembled genome encoded the most frequent variant, which we call the 'wild-type' (Fig. 3c and Supplementary Table S7). Consequences for phase variation were inferred by translating the ORFs and looking for premature stop codons downstream of the repeat tract for each variant. In accounting for potential phase variation, we were better able to recognize (and ultimately adjusted) the start positions of eight proteins (these instances are flagged in Supplementary Table S4).

Analysis of proteins containing TpLRR

We used TMHMM to predict transmembrane helices (v2.0; http://www.cbs.dtu.dk/services/TMHMM/) and SignalP to predict signal peptide cleavage sites (v4.1[40]; http://www.cbs.dtu.dk/services/SignalP/). Protein 3D models were built using Phyre (v2.0[41]; http://www.sbg.bio.ic.ac.uk/phyre2/), which we also used to model other putatively phase variable proteins such as HsdS and Mod (Table S7).

Supplementary Results and Discussion

Metabolic features common to *Ca*. M. girerdii strains

Metabolic reconstructions suggested that *Ca*. M. girerdii strains UC-B3 and VCU-M1 are metabolically identical. See Fettweis *et al*.[23] for an overview.

Ca. M. girerdii is inferred to be glycolytic (sugar-fermenting). All of the enzymes required for glycolysis are present (Supplementary Table S4). Some mycoplasmas also derive energy from arginine via the arginine dihydrolase pathway. We found no evidence of this in *Ca*. M. girerdii, nor any evidence of urea hydrolysis, the metabolism distinguishing *Ureaplasma* spp. A complete phosphotransferase system (PTS) is present. In addition to glucose, it appears to import N-acetylglucosamine (NAG; via nagE) and possibly lactose. Amino sugar (e.g., NAG) catabolism is further supported by the presence of nagA, nagB (two distinct copies), and nanE.

In *Ca*. M. girerdii, the pool of pyruvate (the end-product of glycolysis) may be supplemented by the catabolism (deamination) of serine and alanine. *Ca*. M. girerdii encodes two distinct copies each of serine dehydratase and alanine dehydrogenase. These enzymes are rare among Mollicutes, especially the serine dehydratases, which are known only in *Acholeplasma* spp. and *Ca*. Izimaplasma spp (lineages occupying the taxonomically ambiguous base of the class Mollicutes, which branches *within* the phylum Firmicutes[42-44]).

Conversion of pyruvate to lactate by *Ca*. M. girerdii is unlikely, as strains UC-B3 and VCU-M1 both encode lactate dehydrogenases (LDHs) containing frameshift mutations. Of note, the closest homologs to *Ca*. M. girerdii's LDHs are from *Enterococcus* spp. (~75% amino acid identity), suggesting lateral transfer. As described in the main text, pyuvate decarboxylation to acetyl-CoA is predicted to be carried out by pyruvate formate-lyase (PFL) and/or pyruvate-ferredoxin oxidoreductase (PFOR). Pyruvate dehydrogenase (PDH) was not detected in either *Ca*. M. girerdii genome.

From acetyl-CoA, acetate is formed in two steps via phosphate acetyltransferase and acetate kinase, both of which are present. Ethanol production from acetyl-CoA is also possible, as *Ca*. M. girerdii encodes a bifunctional acetaldehyde/alcohol dehydrogenase (AdhE) and an iron-containing alcohol dehydrogenase. [PFL deactivation (recycling) by AdhE has also been proposed, but this role has been disputed[45].] There is no evidence of a TCA cycle in *Ca*. M. girerdii, nor of any respiratory electron transport (e.g., no quinones or cytrochromes were found). Each strain encodes a complete ATP synthase, which, in this organism, probably consumes ATP to maintain a proton gradient.

Ca. M. girerdii lacks both superoxide dismutase and catalase for the mitigation of oxygen toxicity. The organism does appear to encode superoxide reductase (desulfoferrodoxin), rubredoxin and rubrerythrin. These proteins

belong to an alternative detoxification pathway known in some anaerobes, but not among Mollicutes. Also present are proteins possibly involved in nitric oxide detoxification: these are the electron carriers annotated here as flavorubredoxin and flavohemoprotein. We infer that *Ca*. M. girerdii is not a strict anaerobe.

Ferrous iron uptake is likely facilitated by Feo in *Ca*. M. girerdii. Both strains encode FeoA and FeoB. These proteins are notable because they have not been detected in other mycoplasmas and are known only in a handful of basal Mollicutes (e.g., two *Spiroplasma* spp., *Ca*. Izimaplasma spp., *Acholeplasma brassicae*). Assembly of iron-sulfur clusters is essential to many proteins and *Ca*. M. girerdii encodes a complete set of Suf genes.

In summary, *Ca*. M. girerdii possess anaerobe-like metabolic componentry that is unique among mycoplasmas and more common to members of the phylum Firmicutes, within which the Mollicutes diverged.

Antibiotic resistance in *Ca*. M. girerdii

Lacking cell walls, mycoplasmas are intrinsically resistant to three of the four antibiotics given to the premature infant: the beta-lactams ampicillin and cefotaxime, and the glycopeptide vancomycin. Because gentamicin (the fourth antibiotic given) does not penetrate eukaryotic cells, mycoplasmas can avoid it by invading host cells. Here, possible eukaryotic hosts for *Ca*. M. girerdii included human and *T. vaginalis*. In terms of acquired aminoglycoside resistance, enzymatic modification is most common. *Ca*. M. girerdii encodes a protein exhibiting modest homology to an aminoglycoside phosphotransferase, but this annotation is somewhat speculative (Supplementary Table S4). It also encodes a putative ABC antibiotic efflux pump and a multi-antimicrobial extrusion (MATE) family protein (Supplementary Table S4), which might export gentamicin. We found no other evidence of acquired antibiotic resistance mechanisms in *Ca*. M. girerdii.

Genomic features common to *Ca*. M. girerdii strains

We detected 35 non-coding RNAs in *Ca*. M. girerdii str. UC-B3, which, like str. VCU-M1, contains a single *rrn* (5'-16S-23S-5S) (Table 1, Supplementary Table S4 and Fig. 3a). Apart from 2 SNPs in the 23S rRNA gene, the strains' operons are identical. Both strains contain 31 tRNA genes. In UC-B3, 11 amino acids are represented by a single anticodon, 6 (Gly, Ile, Lys, Ser, Thr, Trp) by 2 anticodons, and 2 (Arg, Leu) by 3 anticodons. Str. UC-B3 encodes 3 distinct tRNAs using the anticodon CAT. These were distinguished as the initiator and elongator Met tRNAs, and the lysylated Ile tRNA. The latter relies on tRNA(Ile)-lysidine synthetase (TilS), which was also detected. Between the strains, 30 of the 31 tRNA gene sequences were identical; only tRNA-Phe-GAA contained a single SNP. Finally, in UC-B3, we located the RNA component of ribonuclease P (as well as the protein component). Two SNPs were found over the homologous region in VCU-M1.

We predicted 574 protein-coding genes in *Ca*. M. girerdii str. UC-B3, a number similar to that found in str. VCU-M1[23] (Table 1 and Supplementary Table S4; the numbers are not directly comparable due to minor differences in

gene prediction strategy between the two studies). Some contained frameshift mutations (n = 9) or internal stop codons (n = 1), or were truncated (n = 4) or fused (n = 1), and may not have been functional (Supplementary Table S4). Most (n = 529) had a syntenic, high-identity (≥ 97% nucleotide identity), reciprocal best hit in VCU-M1 (this includes 13 that mapped closely and syntenically to VCU-M1, but not within, or entirely within ORFs predicted by that study). For these pairs of straightforward orthologs, the average level of nucleotide identity was ~99.7% and the average level of amino acid identity was ~99.5%. The rest (n = 45) were either absent from VCU-M1, divergent from their closest hit in VCU-M1, or appeared in multiple copies in UC-B3. Described in the main text and in detail below, these 45 highlight the basis of strain-level differentiation in this novel, as-yet uncultivated species.

Neither strain was found to encode ribosomal proteins L25, L30 or S1, which are known to have heterogeneous distributions in Bacteria, especially among organisms with reduced genomes[21,46]. We did not identify a plasmid in strain UC-B3 (see Fettweis *et al.*[23]).

CRISPR-Cas systems have been reported in mycoplasmas[47], including in *Ca*. M. girerdii str. VCU-M1[23]. CRISPR arrays are highly dynamic and likely to differ between closely related strains[48]. Also, alteration of the *M. gallisepticum* array has been associated with expansion into a new host species[49]. We wondered whether str. UC-B3 contained a CRISPR-Cas system and, if so, whether it differed from that of VCU-M1.

We searched our dataset using several complementary approaches (see Supplementary Methods) and found substantial portions of a CRISPR-Cas system (of type I-F[50]) in only one bin, our *Enterobacter cloacae* bin (described below). Searching the reported VCU-M1 'CRISPR consensus direct repeat' against the VCU-M1 genome, we found, in 10 of 11 instances, that the repeat actually appeared within genes annotated as BspA-like proteins (described below). In UC-B3, the repeat appears 12 times, all within BspA-like proteins. Therefore, the repeat seems to be associated with the leucine-rich repeat regions characteristic of these proteins (described below), rather than with CRISPR arrays. Neither *Ca*. M. girerdii strain contained any recognizable *cas* genes.

Genomic features distinguishing *Ca*. M. girerdii strain UC-B3 from strain VCU-M1

As noted above, we identified 45 protein-coding genes in str. UC-B3 that did *not* have a syntenic, high-identity (≥ 97% nucleotide identity), reciprocal best hit in strain VCU-M1. We refer to these as UC-B3's 'variable set' (Supplementary Table S4). For those with a valid hit in str. VCU-M1, the average amino acid identity was ~74.1%. Most of these genes (40/45) could be grouped into one of four classes constituting major themes of strain-level differentiation in *Ca*. M. girerdii: (1) diverse BspA-like proteins, (2) multiple truncated copies of fructose-bisphosphate aldolase (FBA) in UC-B3, (3) diverse restriction-modification systems, and (4) a genomic island carrying cytosine methyltransferases in UC-B3 (Fig. 3). Described below, expansion and elaboration in these areas is particularly salient, given *Ca*. M. girerdii's highly reduced genome size and metabolic repertoire:

(1) Diverse BspA-like proteins

In str. UC-B3, we detected 28 distinct proteins containing *Treponema pallidum* leucine-rich repeats (TpLRR; also known as LRR_5) (Supplementary Table S4), a number similar to that reported in str. VCU-M1 (n = 26)[23]. The family of TpLRR-containing proteins includes TpLRR from *T. pallidum*[51], LrrA from *Treponema denticola*[52], BspA from *Tannerella forsythia* (formerly *Bacteroides forsythus*)[53], PcpA from *Streptococcus pneumoniae*[54], the BspA-like proteins of *T. vaginalis*[18], and others. We concur with Fettweis *et al.*[23] that the TpLRR-containing proteins of *Ca*. M. girerdii are most similar to BspA-like proteins, which have been associated with the cell surface and are named for *B. forsythus* surface protein A[53]. BspA-like proteins have been shown to be involved in cell attachment, invasion, aggregation, and in the triggering of host immune responses[52,53,55,56].

Str. UC-B3's 28 TpLRR-containing proteins range in length from 135 – 1,482 amino acids. A signal peptide was detected in 16/28 of them (a larger fraction than in str. VCU-M1[23], largely because in accounting for phase variation, as described in the main text, we localized start sites more consistently). As in VCU-M1, nearly all of UC-B3's BspA-like proteins contain a C-terminal transmembrane helix, and some also contain an N-terminal one. Both genomes encode a secretory pathway for the insertion of membrane proteins, as well as signal peptidase II, which is normally lipoprotein specific but could have a broader function in some mycoplasmas (signal peptidase I like activity has been identified in, e.g., *M. pneumoniae*, which does not contain a conserved sequence encoding the protein[57]). In UC-B3, we detected 3 additional short TpLRR-free proteins that clearly resemble portions of UC-B3 TpLRR-containing proteins, which we annotated as BspA-like 'fragments'. Thus, in total, we designated 31 proteins in UC-B3 as 'BspA-like' (Fig. 3a); their ORFs occupy 7.4% of the genome.

Fifteen of the 45 str. UC-B3 'variable set' genes were BspA-like proteins. On average, their amino acid sequences were 74% identical to the closest homologs in VCU-M1 [range of ≤ 25% (the cutoff) to 96%] (Fig. 3a). Some pairs of homologs appeared to represent simple cases of sequence divergence (i.e., syntenic, low-identity, reciprocal best hits), while others exhibited low-identity hits to non-syntenic and/or partial proteins, or no hit at all. Nucleotide-based mini-alignments of str. UC-B3 to VCU-M1 over regions encoding divergent BspA-like proteins suggest that insertions and deletions of genes, or large fragments thereof, have taken place.

Expansion of gene families is a common theme in the generation of surface variation in pathogens, including in mycoplasmas[58]. That *Ca*. M. girerdii and *T. vaginalis* have expanded the same gene family (TpLRR-containing proteins with similarity to BspA) may reflect niche overlap (binding to epithelial cells), an interaction (co-aggregation), molecular mimicry, or some combination thereof between the two organisms.

(2) Multiple truncated copies of FBA in UC-B3

Fructose-1,6-bisphosphate aldolase (FBA) is a glycolytic enzyme that has been shown to 'moonlight' as a virulence factor[59]. In str. UC-B3, we detected 2 full-length and 3 truncated copies of FBA (Supplementary Table S4). The first full-length copy is nearly identical to its homolog in VCU-M1 (1 synonymous SNP over 885 nt). Presumably, this is the metabolically active copy. The second full-length copy contains frameshift mutations (the 1st is at position 531) and is less similar to its homolog in VCU-M1 (34 SNPs over 886 nt), which also contains frameshift mutations. Within each strain, the two full-length copies are divergent (~60% nucleotide identity).

Another difference between UC-B3 and VCU-M1 is in the number of truncated copies of FBA, which are (or are nearly) duplicates of the last 300 nt of the first full-length copy. In UC-B3, we found 3 truncated copies – 2 that are identical to each other and to the last 300 nt of the first full-length copy, and 1 that is nearly so (2 synonymous SNPs). Indeed, mapping our reads to the first full-length copy alone reveals 3-fold excess coverage over the last 300 nt. Only 1 truncated copy appears in VCU-M1 – it contains 3 synonymous SNPs compared to the last 300 nt of the first full-length copy in VCU-M1. Two of these SNPs are shared with the UC-B3 truncated copy that contains SNPs. In both strains, the first full-length and all truncated copies appear adjacent to BspA-like proteins (end-to-end) within tandem arrays of BspA-like proteins, at two distinct loci. In VCU-M1, a single FBA appears in each array (BspA-FBA), while in UC-B3, two appear (BspA-FBA-BspA-FBA). It seems that FBA may be involved (or simply caught up) in the expansion of BspA-like protein arrays at these two loci.

'Moonlighting' of metabolic enzymes typically involves surface-localization (despite lack of signals or anchors) and a role in adhesion, binding, or immune stimulation[60]. In mycoplasmas, this has been demonstrated for a few such enzymes (but not for FBA); conversely, FBA has been suggested to moonlight in some species, but not in mycoplasmas[59,60]. The reason for multiple copies of FBA, and in particular (multiple) truncated C-terminal copies of FBA, is unknown here and may be unrelated to 'moonlighting' per se. But their co-localization with BspA-like proteins is intriguing.

(3) Diverse R-M systems

Restriction-modification (R-M) systems allow bacteria to protect (by modifying) their own DNA and to degrade (or 'restrict') foreign DNA. They are common among mycoplasmas[61]. In strain UC-B3, we found 18 genes belonging to putative R-M systems (Fig. 3a and Supplementary Table S4), a number similar to that found in strain VCU-M1 (n = 16)[23]. ORFs encoding R-M systems occupy 4% of the UC-B3 genome. Type I, II and III R-M systems were detected, and these vary in the manner and degree to which they distinguish the two strains.

In UC-B3, we found two distinct, type II R-M loci that are highly conserved in sequence and location with the homologous regions of VCU-M1 (Fig. 3a; each locus consists of a methyltransferase and an endonuclease; the second locus is DpnII-like). In UC-B3, we also found two distinct, type III R-M loci, the first of which is conserved and the second of which is inserted with respect to the homologous regions of VCU-M1 [Fig. 3a; again, each locus consists of a methyltransferase (*mod*) and an endonuclease (*res*)]. The insert, carrying the second type III locus and no other genes, is 4.4 kbp in length, interrupts a gene encoding a short BspA-like protein, is flanked by 38-nt direct terminal repeats, and was likely acquired from *Mycoplasma hominis*, although inserted into the methyltransferase is a highly divergent, non-*M. hominis*-like domain. The 38-nt repeat is a portion of the interrupted BspA-like gene; it appears exactly twice in UC-B3 and exactly once in VCU-M1. R-M systems are commonly associated with mobile elements[62,63]. Transposons contain direct terminal repeats; they also contain internal inverted repeats which we did not detect. Nonetheless, it seems clear that UC-B3 acquired (or, less likely, VCU-M1 lost) an *M. hominis*-like type III R-M locus after the two strains diverged. Later, we present evidence that type III R-M systems are likely variably expressed within and between populations of *Ca*. M. girerdii.

Strain UC-B3 encodes 10 genes associated with a type I R-M system. These systems involve three kinds of proteins: a restriction endonuclease (HsdR), modification methyltransferase (HsdM), and DNA specificity subunit (HsdS). HsdM and HsdS are sufficient for methylation activity. Strain UC-B3 contains one type I R-M locus in which two specificity genes (*hsdS*; 1 intact, 1 truncated) are flanked by an upstream methyltransferase (*hsdM*) and a downstream endonuclease (*hsdR*) (Fig. 3a). UC-B3 also contains a second type I R-M locus, lacking *hsdR*, in which *hsdM* is again situated upstream of a complex arrangement of *hsdS* (one intact gene and an adjacent fragment; phase variation likely affects *hsdS* at both loci, as described below). The two HsdM proteins are not similar (~19% amino acid identity) and probably belong to different families (unlike in the murine pathogen *Mycoplasma pulmonis*, for example, where the loci are similar[64]). Dispersed throughout the UC-B3 genome are four additional orphan *hsdS*, for a total of seven distinct genes, a number not unusually high among mycoplasmas (e.g., *Mycoplasma pneumoniae* encodes 10 *hsdS* genes[65]).

In general, HsdS proteins consist of two target recognition domains (TRDs) separated by a central conserved region and flanked by distal conserved regions. The TRDs dictate DNA sequence specificity. In UC-B3, five of the HsdS proteins have central conserved regions bearing tandem repeats of the tetra-amino-acid 'KAEL' in varying numbers: two have two repeats, two have five, and one has seven. These repeats, characteristic of type IC R-M systems, appear to be species-specific (e.g., *M. pneumoniae* uses 'SAEL'[65]) and can affect complementation[66]. The length of the repeat tract can also affect DNA specificity[67]. In terms of TRDs, UC-B3's HsdS proteins comprise a remarkably diverse array. Together, these features likely underpin a dynamic,

adaptable system for carrying out methylation-associated tasks, from defense against foreign DNA to the regulation of gene expression[66-69].

R-M systems contribute substantially to strain-level diversity in *Ca*. M. girerdii. Eleven of UC-B3's 45 'variable' genes belong to R-M systems. Two of these constitute the inserted type III R-M locus, and the rest belong to type I R-M loci. Although the number, arrangement, and genomic location of genes associated with type I R-M systems is similar between UC-B3 and VCU-M1, the sequences themselves are quite distinct; in part, this appears to be a result of recombination among HsdS TRDs within strains (e.g., the large-scale inversion shown in Fig. 1b and Supplementary Fig. S3 is flanked by *hsdS* genes). Between strains, the average amino acid identity for homologs associated with type I R-M systems is 72% (39-99%), with the lowest observed for the endonuclease HsdR. Like UC-B3, VCU-M1 encodes seven HsdS proteins, five of which contain central conserved regions bearing tandem repeats of the tetra-amino-acid 'KAEL'; however, among them, the number and distribution of repeats differs: one has two repeats, two have four, one has five, and one has six. Strain-level variation involving components of R-M systems is common among host-associated commensals and pathogens (e.g., refs.[65,70,71]). An open question pertains to the nature and relative strengths of forces driving R-M system diversification – whether this fine-scale variation is driven primarily by a need to protect against diverse and dynamic sources of foreign DNA (e.g., phage) or to vary gene expression.

(4) Genomic island carrying cytosine methyltransferases in UC-B3

DNA adenines are the predicted target of the six methyltransferases associated with strain UC-B3's R-M systems. UC-B3 encodes four additional methyltransferases that are predicted to target DNA cytosines (C-5 MTases) and are seemingly unassociated with R-M systems. The 418-aa sequence of one of them is 100% identical to its syntenic homolog, the sole C-5 MTase in strain VCU-M1. Neighboring this locus in both strains are two notable features. First, immediately upstream (and out of frame) is a region encoding a cro/C1-type helix-turn-helix (HTH) DNA-binding domain; indeed, some C-5 MTases contain such a domain, a putative transcriptional regulator, at their N-terminus[72], although it is unlinked here, possibly due to a frameshift mutation. Second, ~1 kbp upstream lies an ~1-kbp region that is highly similar to *M. hominis* (95% nucleotide identity); this region encodes an integrase core domain-containing protein (annotated as an IS1202-like transposase in VCU-M1). In UC-B3, these features are reflected and repeated on a distant 8.6-kbp fragment, inserted with respect to VCU-M1 (between *secA* and *holA*), bearing three additional, distinct C-5 MTases (Fig. 3a,b and Supplementary Table S4). We refer to this insert as a genomic island (Fig. 3a,b).

Of the 45 'variable' genes in UC-B3, 10 are on the 8.6-kbp genomic island (Fig. 3b). Highly similar to genome sequences from *M. hominis* (98-99% nucleotide identity), the island contains two nearly identical integrase-like proteins (putative transposases). Oriented similarly, the first, located near the center of the

island, is intact, while the second, located at the *holA* end, is truncated. Comparison over 823 nt reveals 10 SNPs (5 synonymous, 5 nonsynonymous) and one insertion (1 nt; inducing a late frameshift in the truncated copy). Between these integrases, which are distinct from the integrase core domain-containing protein found at the first locus, lie two hypothetical proteins and a 520-aa C-5 MTase bearing an N-terminal cro/C1-type HTH domain. The nucleotide sequence of this C-5 MTase has been observed in its entirety in only two other genomes, both in draft status. They are from *M. hominis* isolates PL5 from the plancenta of a woman in spontaneous preterm labor (isolated in the early 1990s)[73] and H34 from an infected abdominal incision following hysterectomy (isolated before 1962)[74]. The other two island-associated C-5 MTases lie upstream of the area bounded by the integrases. The first is intact and identical (100% over 325 aa) to an *M. hominis* modification methylase. No cro/C1-type HTH domain exists in or near this gene. The second is truncated to 80 amino acids due in part to a frameshift mutation, and is probably nonfunctional. All four of UC-B3's C-5 MTases are distinct.

The structure of the genomic island is suggestive of a transposable element, but results of searches for, e.g., specific insertion sequences and direct and inverted repeats were ambiguous. A streptococcal transposon carrying a gene encoding a C-5 MTase has been reported[75].

In summary, despite its phylogenetic and metabolic novelty, *Ca*. M. girerdii exhibits strain-level diversity that appears to involve strategies and features similar to those driving differentiation within other *Mycoplasma* spp.[58] and more broadly, within other host-associated commensals and pathogens (e.g., references [70,76,77]).

Indel sequencing error is an unlikely source of variations in frame

Variations in frame are caused by insertions and deletions (indels). Indels may arise by natural mutation or by artifact such as sequencing error. Here, we suggest that the observed indels in population *Ca*. M. girerdii str. UC-B3 were more likely to have arisen by mutation. There are several lines of evidence supporting this claim.

First, the frequencies we observed were orders of magnitude higher than expected for sequencing error. Our sequence data were generated on the Illumina HiSeq platform. On this platform, indel error rates have been shown to be quite low, indeed, much lower than substitution error rates. For example, Schirmer *et al.*[78] reported, in a comprehensive and systematic study of Illumina sequencing errors based on a mock community, per-base insertion and deletion error rates of 0.0000028 and 0.0000051, respectively, for read 1, and 0.0000035 and 0.0000049, respectively, for read 2. It seems reasonable to suggest that these rates likely reflect the chances that for a given base in the UC-B3 genome, an observed indel had been introduced by sequencing error. In this study, we found frequencies ranging from 0.017 to 0.446 for variants resulting from indels at specific sites. These frequencies are much higher than expected for indel sequencing error.

There are several other reasons why we think it's unlikely that sequencing error alone explains our data, consisting largely of dinucleotide indels at specific dinucleotide repeat tracts. First, to minimize the impacts of sequencing error, we discarded low quality data by filtering and trimming our reads. Second, the indels we observed were stereotyped to dinucleotides matching the tracts; for those introduced by sequencing error, we might expect a different distribution, likely skewed toward single nucleotides of any type of base (A, T, G or C). Third, because of our relatively small insert size, many of the indels we observed were apparent on both paired reads; indeed, whenever this was the case, the pairs matched. It seems extremely unlikely that the same sequencing indel error would arise twice at the same location. Considering the above, we concluded that biological mechanisms such as slipped-strand mispairing better explained the stereotyped, high-frequency indels we observed than did sequencing error.

Detection of CRISPR-Cas system in *Enterobacter cloacae*

Elements indicative of a CRISPR-Cas system were detected in our *Enterobacter* partial genome (Table 1). *Enterobacter*-related Cas genes, including fragments of *cas1, cas3, csy1, csy2, csy3* and *cas6/csy4*, were distributed over eight scaffolds (ranging in length from 327 to 1732 bp), two of which were binned while the others fell below the 1-kbp minimum length threshold set for the ESOM. The binned scaffolds encoded fragments of *cas1, csy1* and *csy2* (Supplementary Table S3 bin4). While the full operon structure remains unclear to us due to the fragmentary nature of the partial genome, the presence of *cas3* and *csy1* suggests a CRISPR-Cas system of subtype I-F (Ypest or CASS3) (*sensu* Makarova *et al.*[50]).

We detected CRISPR arrays on four *Enterobacter* scaffolds. A 28-bp direct repeat TTTCTAAGCTGCCTGTACGGCAGTGAAC (DR1) was exclusive to two scaffolds, while a similar 28-nt direct repeat TTTCTAAGCTGCCTGTACGGCAGAGCAC (DR2) was exclusive to the others. DR1 and DR2 differ by two SNPs, suggesting that at least two arrays were present. One DR1-containing scaffold also encoded the 5' end of *cas1*. Other than these four scaffolds, no others in our assembly contained DR1 or DR2. However, after removing from the read pool all reads mapping to the four DR-containing scaffolds, we could still find (and, to a limited degree, assemble) residual reads containing DR1 or DR2. These new scaffolds shared some but not all spacers with the initial four, suggesting the co-existence of a minor (low-abundance) strain. Unfortunately, further analysis was precluded by extremely low coverage. Indeed, low coverage and fine-scale diversity likely conspire to underpin the high level of fragmentation observed for our *Enterobacter* genome assembly.

We found 33 and 13 spacers associated with DR1 and DR2, respectively. All 46 of these were 32-33 bp in length and unique. We screened our dataset for spacer targets (e.g., phage, plasmids) by identifying reads containing spacers but not DR1 or DR2, and then mapping the identified reads back to our scaffolds. This analysis disclosed the putative target of one DR1-associated spacer—a region encoding the alpha subunit of succinyl-CoA synthetase (*sucD*; 100% match) on a scaffold binned to *Enterobacter*. The reads' pairs also mapped within this

gene or, as in one case, to *sucC* on a presumably adjacent scaffold. This target accounted for all spacer matches to reads. Such apparent self-targeting ('autoimmunity') is paradoxical, but not uncommon. Interestingly, it has been reported that an intact *Chlorobium* CRISPR array also targets a *suc* gene (*sucC*, the beta subunit)[79]. The purpose of self-targeting (if any) is unknown, but may include gene regulation, repair, or recombination, via as-yet-unknown mechanisms[80].

Finally, a search of the NCBI nucleotide database (nr/nt) suggests that one DR2-associated spacer targets (97% match) the genome of Shigella phage Sf6. This spacer was recently reported within a CRISPR array in the genome of *Enterobacter cloacae* complex 'Hoffmann cluster IV' strain DSM 16690 (accession no. CP017184; 100% match). The targets of the other 44 spacers remain unknown to us.

Supplementary References

1. Langmead, B. & Salzberg, S. L. Fast gapped-read alignment with Bowtie 2. *Nat. Methods* **9,** 357–359 (2012).
2. Peng, Y., Leung, H. C. M., Yiu, S. M. & Chin, F. Y. L. IDBA-UD: a *de novo* assembler for single-cell and metagenomic sequencing data with highly uneven depth. *Bioinformatics* **28,** 1420–1428 (2012).
3. Hyatt, D., LoCascio, P. F., Hauser, L. J. & Uberbacher, E. C. Gene and translation initiation site prediction in metagenomic sequences. *Bioinformatics* **28,** 2223–2230 (2012).
4. Suzek, B. E., Huang, H., McGarvey, P., Mazumder, R. & Wu, C. H. UniRef: comprehensive and non-redundant UniProt reference clusters. *Bioinformatics* **23,** 1282–1288 (2007).
5. Edgar, R. C. Search and clustering orders of magnitude faster than BLAST. *Bioinformatics* **26,** 2460–2461 (2010).
6. Lagesen, K. *et al.* RNAmmer: consistent and rapid annotation of ribosomal RNA genes. *Nucleic Acids Res.* **35,** 3100–3108 (2007).
7. Miller, C. S., Baker, B. J., Thomas, B. C., Singer, S. W. & Banfield, J. F. EMIRGE: reconstruction of full-length ribosomal genes from microbial community short read sequencing data. *Genome Biol.* **12,** R44 (2011).
8. Dick, G. J. *et al.* Community-wide analysis of microbial genome sequence signatures. *Genome Biol.* **10,** R85 (2009).
9. Ultsch, A. & Mörchen, F. *ESOM-Maps: tools for clustering, visualization, and classification with Emergent SOM*. (Technical Report Dept. of Mathematics and Computer Science, University of Marburg, Germany, 2005).
10. Caporaso, J. G. *et al.* QIIME allows analysis of high-throughput community sequencing data. *Nat. Methods* **7,** 335–336 (2010).
11. Overbeek, R. *et al.* The SEED and the Rapid Annotation of microbial genomes using Subsystems

Technology (RAST). *Nucleic Acids Res.* **42,** D206–14 (2014).

12. Brettin, T. *et al.* RASTtk: a modular and extensible implementation of the RAST algorithm for building custom annotation pipelines and annotating batches of genomes. *Sci. Rep.* **5,** 8365 (2015).
13. Skennerton, C. T., Imelfort, M. & Tyson, G. W. Crass: identification and reconstruction of CRISPR from unassembled metagenomic data. *Nucleic Acids Res.* **41,** e105 (2013).
14. Finn, R. D., Clements, J. & Eddy, S. R. HMMER web server: interactive sequence similarity searching. *Nucleic Acids Res.* **39,** W29–37 (2011).
15. Gibson, M. K., Forsberg, K. J. & Dantas, G. Improved annotation of antibiotic resistance determinants reveals microbial resistomes cluster by ecology. *The ISME Journal* **9,** 207–216 (2015).
16. Brown, C. T. *et al.* Genome resolved analysis of a premature infant gut microbial community reveals a *Varibaculum cambriense* genome and a shift towards fermentation-based metabolism during the third week of life. *Microbiome* **1,** 30 (2013).
17. Parks, D. H., Imelfort, M., Skennerton, C. T., Hugenholtz, P. & Tyson, G. W. CheckM: assessing the quality of microbial genomes recovered from isolates, single cells, and metagenomes. *Genome Research* **25,** 1043–1055 (2015).
18. Carlton, J. M. *et al.* Draft genome sequence of the sexually transmitted pathogen *Trichomonas vaginalis*. *Science* **315,** 207–212 (2007).
19. Cornelius, D. C. *et al.* Genetic characterization of *Trichomonas vaginalis* isolates by use of multilocus sequence typing. *J. Clin. Microbiol.* **50,** 3293–3300 (2012).
20. Kearse, M. *et al.* Geneious Basic: an integrated and extendable desktop software platform for the organization and analysis of sequence data. *Bioinformatics* **28,** 1647–1649 (2012).
21. Brown, C. T. *et al.* Unusual biology across a group comprising more than 15% of domain Bacteria. *Nature* **523,** 208–211 (2015).
22. Sharon, I. & Banfield, J. F. Genomes from metagenomics. *Science* **342,** 1057–1058 (2013).
23. Fettweis, J. M. *et al.* An emerging mycoplasma associated with trichomoniasis, vaginal infection and disease. *PLoS ONE* **9,** e110943 (2014).
24. Boetzer, M. & Pirovano, W. Toward almost closed genomes with GapFiller. *Genome Biol.* **13,** R56 (2012).
25. Nurk, S. *et al.* Assembling single-cell genomes and mini-metagenomes from chimeric MDA products. *J. Comput. Biol.* **20,** 714–737 (2013).
26. Mackiewicz, P., Zakrzewska-Czerwinska, J., Zawilak, A., Dudek, M. R. & Cebrat, S. Where does bacterial replication start? Rules for predicting the *oriC* region. *Nucleic Acids Res.* **32,** 3781–3791 (2004).
27. Kurtz, S. *et al.* Versatile and open software for comparing large genomes. *Genome Biol.* **5,** R12 (2004).
28. Ondov, B. D. *et al.* Mash: fast genome and metagenome distance estimation using MinHash. *Genome Biol.* **17,** 132 (2016).
29. Kanehisa, M., Sato, Y. & Morishima, K. BlastKOALA and GhostKOALA: KEGG tools for functional characterization of genome and metagenome sequences. *J. Mol. Biol.* **428,** 726–731 (2016).

30. Jones, P. *et al.* InterProScan 5: genome-scale protein function classification. *Bioinformatics* **30,** 1236–1240 (2014).

31. Quast, C. *et al.* The SILVA ribosomal RNA gene database project: improved data processing and web-based tools. *Nucleic Acids Res.* **41,** D590–6 (2013).

32. Lowe, T. M. & Eddy, S. R. tRNAscan-SE: a program for improved detection of transfer RNA genes in genomic sequence. *Nucleic Acids Res.* **25,** 955–964 (1997).

33. Tåquist, H., Cui, Y. & Ardell, D. H. TFAM 1.0: an online tRNA function classifier. *Nucleic Acids Res.* **35,** W350–3 (2007).

34. Brown, J. W. The Ribonuclease P Database. *Nucleic Acids Res.* **27,** 314 (1999).

35. Li, Y. & Altman, S. In search of RNase P RNA from microbial genomes. *RNA* **10,** 1533–1540 (2004).

36. Caspi, R. *et al.* The MetaCyc database of metabolic pathways and enzymes and the BioCyc collection of Pathway/Genome Databases. *Nucleic Acids Res.* **42,** D459–71 (2014).

37. Markowitz, V. M. *et al.* IMG 4 version of the integrated microbial genomes comparative analysis system. *Nucleic Acids Res.* **42,** D560–7 (2014).

38. White, D. *The physiology and biochemistry of prokaryotes*. (Oxford University Press, 1995).

39. Mudunuri, S. B. & Nagarajaram, H. A. IMEx: Imperfect Microsatellite Extractor. *Bioinformatics* **23,** 1181–1187 (2007).

40. Petersen, T. N., Brunak, S., Heijne, von, G. & Nielsen, H. SignalP 4.0: discriminating signal peptides from transmembrane regions. *Nat. Methods* **8,** 785–786 (2011).

41. Kelley, L. A., Mezulis, S., Yates, C. M., Wass, M. N. & Sternberg, M. J. E. The Phyre2 web portal for protein modeling, prediction and analysis. *Nat. Protoc.* **10,** 845–858 (2015).

42. Davis, J. J., Xia, F., Overbeek, R. A. & Olsen, G. J. Genomes of the class *Erysipelotrichia* clarify the firmicute origin of the class *Mollicutes*. *Int. J. Syst. Evol. Microbiol.* **63,** 2727–2741 (2013).

43. Skennerton, C. T. *et al.* Phylogenomic analysis of *Candidatus* 'Izimaplasma' species: free-living representatives from a *Tenericutes* clade found in methane seeps. *The ISME Journal* **10,** 2679–2692 (2016).

44. Hug, L. A. *et al.* A new view of the tree of life. *Nat. Microbiol.* **1,** 16048 (2016).

45. Nnyepi, M. R., Peng, Y. & Broderick, J. B. Inactivation of *E. coli* pyruvate formate-lyase: role of AdhE and small molecules. *Arch. Biochem. Biophys.* **459,** 1–9 (2007).

46. Lecompte, O., Ripp, R., Thierry, J.-C., Moras, D. & Poch, O. Comparative analysis of ribosomal proteins in complete genomes: an example of reductive evolution at the domain scale. *Nucleic Acids Res.* **30,** 5382–5390 (2002).

47. Burstein, D. *et al.* Major bacterial lineages are essentially devoid of CRISPR-Cas viral defence systems. *Nat. Commun.* **7,** 10613 (2016).

48. Sun, C. L., Thomas, B. C., Barrangou, R. & Banfield, J. F. Metagenomic reconstructions of bacterial CRISPR loci constrain population histories. *The ISME Journal* **10,** 858–870 (2016).

49. Delaney, N. F. *et al.* Ultrafast evolution and loss of CRISPRs following a host shift in a novel wildlife pathogen, *Mycoplasma gallisepticum*. *PLoS Genet.* **8,** e1002511 (2012).

50. Makarova, K. S. *et al.* An updated evolutionary classification of CRISPR-Cas systems. *Nat. Rev. Micro.* **13,** 722–736 (2015).

51. Shevchenko, D. V. *et al.* Molecular characterization and cellular localization of TpLRR, a processed leucine-rich repeat protein of *Treponema pallidum*, the syphilis spirochete. *J. Bacteriol.* **179,** 3188–3195 (1997).

52. Ikegami, A., Honma, K., Sharma, A. & Kuramitsu, H. K. Multiple functions of the leucine-rich repeat protein LrrA of *Treponema denticola*. *Infect. Immun.* **72,** 4619–4627 (2004).

53. Sharma, A. *et al.* Cloning, expression, and sequencing of a cell surface antigen containing a leucine-rich repeat motif from *Bacteroides forsythus* ATCC 43037. *Infect. Immun.* **66,** 5703–5710 (1998).

54. Sanchez-Beato, A. R., Lopez, R. & García, J. L. Molecular characterization of PcpA: a novel choline-binding protein of *Streptococcus pneumoniae*. *FEMS Microbiology Letters* **164,** 207–214 (1998).

55. Inagaki, S., Onishi, S., Kuramitsu, H. K. & Sharma, A. *Porphyromonas gingivalis* vesicles enhance attachment, and the leucine-rich repeat BspA protein is required for invasion of epithelial cells by "*Tannerella forsythia*". *Infect. Immun.* **74,** 5023–5028 (2006).

56. de Miguel, N. *et al.* Proteome analysis of the surface of *Trichomonas vaginalis* reveals novel proteins and strain-dependent differential expression. *Mol. Cell Proteomics* **9,** 1554–1566 (2010).

57. Catrein, I., Herrmann, R., Bosserhoff, A. & Ruppert, T. Experimental proof for a signal peptidase I like activity in *Mycoplasma pneumoniae*, but absence of a gene encoding a conserved bacterial type I SPase. *FEBS J.* **272,** 2892–2900 (2005).

58. Razin, S., Yogev, D. & Naot, Y. Molecular biology and pathogenicity of mycoplasmas. *Microbiol. Mol. Biol. R.* **62,** 1094–1156 (1998).

59. Shams, F., Oldfield, N. J., Wooldridge, K. G. & Turner, D. P. J. Fructose-1,6-bisphosphate aldolase (FBA)-a conserved glycolytic enzyme with virulence functions in bacteria: 'ill met by moonlight'. *Biochem. Soc. Trans.* **42,** 1792–1795 (2014).

60. Pancholi, V. & Chhatwal, G. S. Housekeeping enzymes as virulence factors for pathogens. *Int. J. Med. Microbiol.* **293,** 391–401 (2003).

61. Brocchi, M., Vasconcelos, A. T. R. de & Zaha, A. Restriction-modification systems in *Mycoplasma* spp. *Genet. Mol. Biol.* (2007).

62. Furuta, Y., Abe, K. & Kobayashi, I. Genome comparison and context analysis reveals putative mobile forms of restriction-modification systems and related rearrangements. *Nucleic Acids Res.* **38,** 2428–2443 (2010).

63. Oliveira, P. H., Touchon, M. & Rocha, E. P. C. The interplay of restriction-modification systems with mobile genetic elements and their prokaryotic hosts. *Nucleic Acids Res.* **42,** 10618–10631 (2014).

64. Sitaraman, R. & Dybvig, K. The *hsd* loci of *Mycoplasma pulmonis*: organization, rearrangements and

expression of genes. *Mol. Microbiol.* **26,** 109–120 (1997).

65. Xiao, L. *et al.* Comparative genome analysis of *Mycoplasma pneumoniae*. *BMC Genomics* **16,** 610–610 (2015).

66. Adamczyk-Popławska, M., Kondrzycka, A., Urbanek, K. & Piekarowicz, A. Tetra-amino-acid tandem repeats are involved in HsdS complementation in type IC restriction-modification systems. *Microbiology* **149,** 3311–3319 (2003).

67. Furuta, Y. *et al.* Methylome diversification through changes in DNA methyltransferase sequence specificity. *PLoS Genet.* **10,** e1004272 (2014).

68. Gubler, M., Braguglia, D., Meyer, J., Piekarowicz, A. & Bickle, T. A. Recombination of constant and variable modules alters DNA sequence recognition by type IC restriction-modification enzymes. *EMBO J.* **11,** 233–240 (1992).

69. Srikhanta, Y. N., Fox, K. L. & Jennings, M. P. The phasevarion: phase variation of type III DNA methyltransferases controls coordinated switching in multiple genes. *Nat. Rev. Micro.* **8,** 196–206 (2010).

70. Xu, Q., Morgan, R. D., Roberts, R. J. & Blaser, M. J. Identification of type II restriction and modification systems in *Helicobacter pylori* reveals their substantial diversity among strains. *Proc. Natl. Acad. Sci. USA* **97,** 9671–9676 (2000).

71. Croucher, N. J. *et al.* Diversification of bacterial genome content through distinct mechanisms over different timescales. *Nat. Commun.* **5,** 5471–5471 (2014).

72. Casadesús, J. & Low, D. Epigenetic gene regulation in the bacterial world. *Microbiol. Mol. Biol. R.* **70,** 830–856 (2006).

73. Allen-Daniels, M. J. *et al.* Identification of a gene in *Mycoplasma hominis* associated with preterm birth and microbial burden in intraamniotic infection. *Am. J. Obstet. Gynecol.* **212,** 779.e1–779.e13 (2015).

74. Lemcke, R. & Csonka, G. W. Antibodies against pleuropneumonia-like organisms in patients with salpingitis. *Br. J. Vener. Dis.* **38,** 212–217 (1962).

75. Sampath, J. & Vijayakumar, M. N. Identification of a DNA cytosine methyltransferase gene in conjugative transposon Tn5252. *Plasmid* **39,** 63–76 (1998).

76. Tunio, S. A. *et al.* The moonlighting protein fructose-1, 6-bisphosphate aldolase of *Neisseria meningitidis*: surface localization and role in host cell adhesion. *Mol. Microbiol.* **76,** 605–615 (2010).

77. Manuel, C. S., Van Stelten, A., Wiedmann, M., Nightingale, K. K. & Orsi, R. H. Prevalence and distribution of *Listeria monocytogenes inlA* alleles prone to phase variation and *inlA* alleles with premature stop codon mutations among human, food, animal, and environmental isolates. *Appl. Environ. Microbiol.* **81,** 8339–8345 (2015).

78. Schirmer, M., D'Amore, R., Ijaz, U. Z., Hall, N. & Quince, C. Illumina error profiles: resolving fine-scale variation in metagenomic sequencing data. *BMC Bioinformatics* **17,** 125 (2016).

79. Stern, A., Keren, L., Wurtzel, O., Amitai, G. & Sorek, R. Self-targeting by CRISPR: gene regulation or autoimmunity? *Trends Genet.* **26,** 335–340 (2010).

80. Westra, E. R., Buckling, A. & Fineran, P. C. CRISPR-Cas systems: beyond adaptive immunity. *Nat. Rev. Micro.* **12,** 317–326 (2014).

Supplementary Figure Legends

Supplementary Figure S1. Preview of microbial genome bins. (a) Rank abundance of prospective genomes estimated from an inventory of ribosomal proteins (RPs). RPs were grouped according to organismal taxonomic assignment. Plotted for each group (taxon) is the average (± SD) coverage of RP-bearing scaffolds. Counts appear in parentheses (no. of scaffolds, no. of RPs) and the taxonomic level (genus or family) reflects the consensus assignment. All RPs were counted, including duplicates and partials. **(b)** Bin exploration using two genomic signatures—coverage and %G+C—for scaffolds >10 kb (large coral circles) and 1-10 kb (small gray circles) in size. Clustering evident among larger scaffolds (suggesting three bins) is obscured in the presence of the smaller ones.

Supplementary Figure S2. Binning scaffolds to microbial genomes. Primary binning was carried out using a tetranucleotide frequency-based emergent self-organizing map (ESOM). Each point on the map corresponds to a fragment of either a reference genome (‘Reference’) or *de novo* assembled scaffold (‘Unknown’). Clusters (putative bins), bounded by darker areas of the map, contain fragments with similar tetranucleotide frequencies. Selection of reference genomes was guided by the gene inventory shown in Supplementary Fig. S1a. All *de novo* assembled scaffolds ≥ 1 kb were included in the analysis (n = 1,688). Fragment length ranged from 1,000 to 9,951 bp (4,520 ± 1,370 bp). The map is periodic and the white box outlines one interval. Numbers 1-6 correspond to the curated bins shown in Table 1: bin 1, *Pseudomonas*; bin 2, *Mycoplasma*; bin 3, *Streptococcus*; bin 4, *Enterobacter*; bin 5, *Staphylococcus*; bin 6, *Trichomonas*. The genome of *Ca*. M. girerdii str. VCU-M1 was not included as a reference because it was not available at the time that we created and analyzed the ESOM.

Supplementary Figure S3. Dotplot for alignment of finished *Ca*. M. girerdii genomes. NUCmer was used to align the finished genome of *Ca*. M. girerdii strain UC-B3 (this study) against that of *Ca*. M. girerdii strain VCU-M1[23]. Displayed are nucleotide alignment blocks ≥ 66 bp in length with ≥ 80% sequence identity. Forward and reverse matches are shown in purple and blue, respectively.

Supplementary Figure S4. *Mycoplasma* phylogeny including *Ca*. M. girerdii strains. Maximum likelihood phylogeny inferred from an alignment of ribosomal protein S3 amino acid sequences. Bootstrap values > 50% are displayed. The scale bar represents 0.5 substitutions per site. NCBI accession numbers are shown in parentheses.

Supplementary Tables

Supplementary Table S1. Relative abundance of taxa detected using 16S rRNA gene surveys, weighted by the amount of DNA combined for metagenomic sequencing, for the premature infant's oral samples

Supplementary Table S2. Ribosomal RNA genes predicted on *de novo* assembled scaffolds using RNAmmer

Supplementary Table S3. Preliminary annotation of bins reconstructed from the oral metagenome of a 3-week-old premature infant

Supplementary Table S4. Final annotation of finished genome of *Ca*. M. girerdii str. UC-B3 reconstructed from the oral metagenome of a 3-week-old premature infant

Supplementary Table S5. Presence/absence of genes involved in energy metabolism across Mollicutes and close relatives including *Ca*. M. girerdii strains UC-B3 and VCU-M1

Supplementary Table S6. Perfect dinucleotide tandem repeats $\geq$ 5 iterations in length in genome of *Ca*. M. girerdii str. UC-B3

Supplementary Table S7. Length variation in DNA tandem repeats, revealed using metagenomics, suggests possible phase variation within population *Ca*. M. girerdii str. UC-B3 colonizing the oral cavity of a premature infant

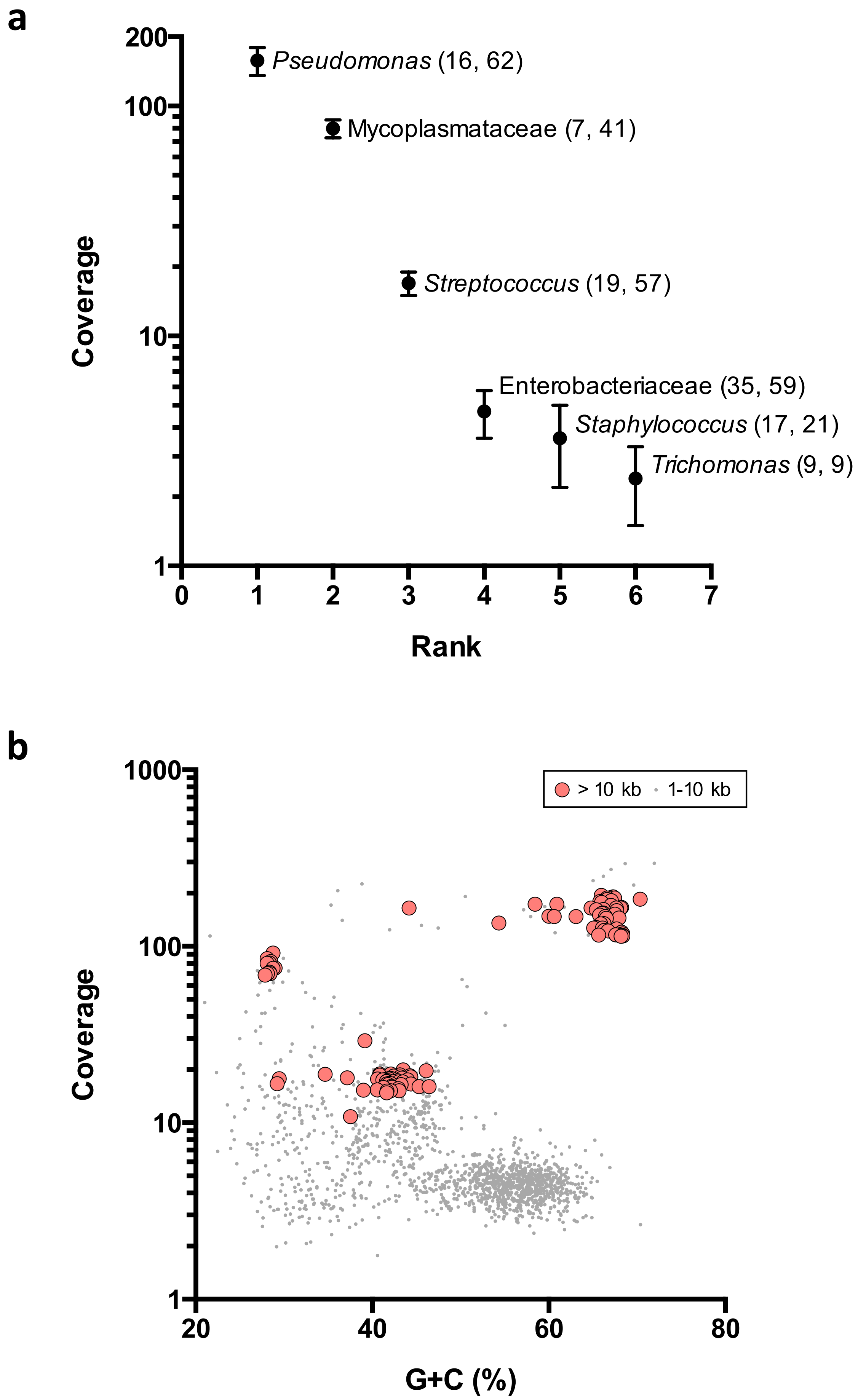

Supplementary Figure S1. Preview of microbial genome bins. (a) Rank abundance of prospective genomes estimated from an inventory of ribosomal proteins (RPs). RPs were grouped according to organismal taxonomic assignment. Plotted for each group (taxon) is the average (± SD) coverage of RP-bearing scaffolds. Counts appear in parentheses (no. of scaffolds, no. of RPs) and the taxonomic level (genus or family) reflects the consensus assignment. All RPs were counted, including duplicates and partials. **(b)** Bin exploration using two genomic signatures—coverage and %G+C—for scaffolds >10 kb (large coral circles) and 1-10 kb (small gray circles) in size. Clustering evident among larger scaffolds (suggesting three bins) is obscured in the presence of the smaller ones.

Figure S1

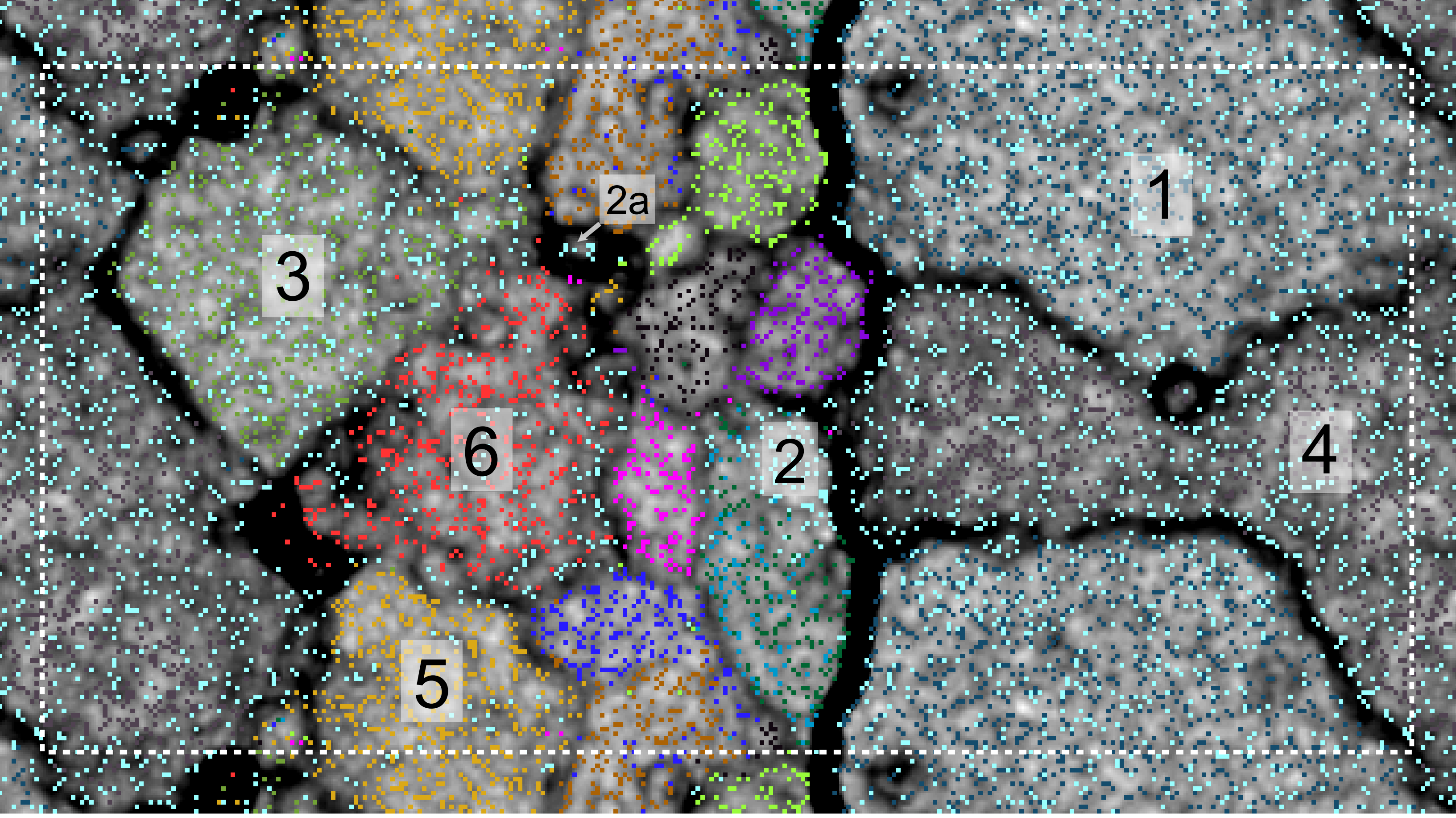

Unknown:

- Premature infant oral metagenome

Reference:

- *Enterobacter cloacae* subsp. cloacae NCTC 9394, draft genome NC_021046
- *Mycoplasma gallisepticum* str. R(low), complete genome NC_004829
- *Mycoplasma genitalium* G37, complete genome NC_000908
- *Mycoplasma hominis* ATCC 23114, complete genome NC_013511
- *Mycoplasma iowae* 695, draft genome AGFP01000000
- *Mycoplasma penetrans* HF-2, complete genome NC_004432
- *Mycoplasma pneumoniae* M129, complete genome NC_000912
- *Pseudomonas aeruginosa* YL84, complete genome CP007147
- *Staphylococcus epidermidis* ATCC 12228, complete genome NC_004461
- *Streptococcus parasanguinis* FW213, complete genome NC_017905
- *Trichomonas vaginalis* G3, scaffolds NW_001820792-6 (the 5 largest scaffolds)
- *Ureaplasma parvum* serovar 3 str. ATCC 27815, complete genome NC_010503
- *Ureaplasma urealyticum* serovar 10 str. ATCC 33699, complete genome NC_011374

Supplementary Figure S2. Binning scaffolds to microbial genomes. Primary binning was carried out using a tetranucleotide frequency-based emergent self-organizing map (ESOM). Each point on the map corresponds to a fragment of either a reference genome ('Reference') or *de novo* assembled scaffold ('Unknown'). Clusters (putative bins), bounded by darker areas of the map, contain fragments with similar tetranucleotide frequencies. Selection of reference genomes was guided by the gene inventory shown in Supplementary Fig. S1a. All *de novo* assembled scaffolds ≥ 1 kb were included in the analysis (n = 1,688). Fragment length ranged from 1,000 to 9,951 bp (4,520 ± 1,370 bp). The map is periodic and the white box outlines one interval. Numbers 1-6 correspond to the curated bins shown in Table 1: bin 1, *Pseudomonas*; bin 2, *Mycoplasma*; bin 3, *Streptococcus*; bin 4, *Enterobacter*; bin 5, *Staphylococcus*; bin 6, *Trichomonas*. The genome of *Ca*. M. girerdii str. VCU-M1 was not included as a reference because it was not available at the time that we created and analyzed the ESOM.

Figure S2

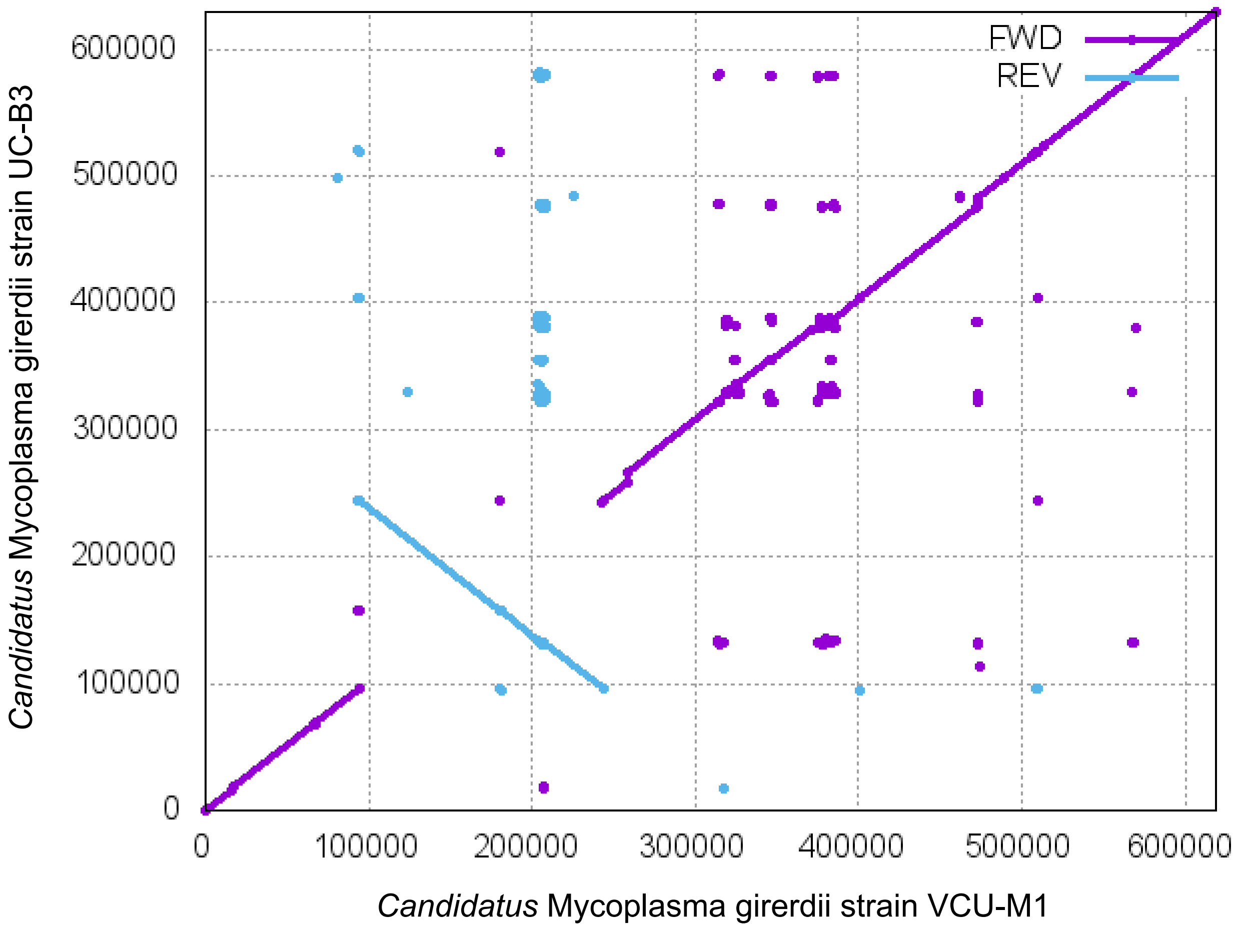

Supplementary Figure S3. Dotplot for alignment of finished *Ca*. M. girerdii genomes. NUCmer was used to align the finished genome of *Ca*. M. girerdii strain UC-B3 (this study) against that of *Ca*. M. girerdii strain VCU-M1[23]. Displayed are nucleotide alignment blocks ≥ 66 bp in length with ≥ 80% sequence identity. Forward and reverse matches are shown in purple and blue, respectively.

Figure S3

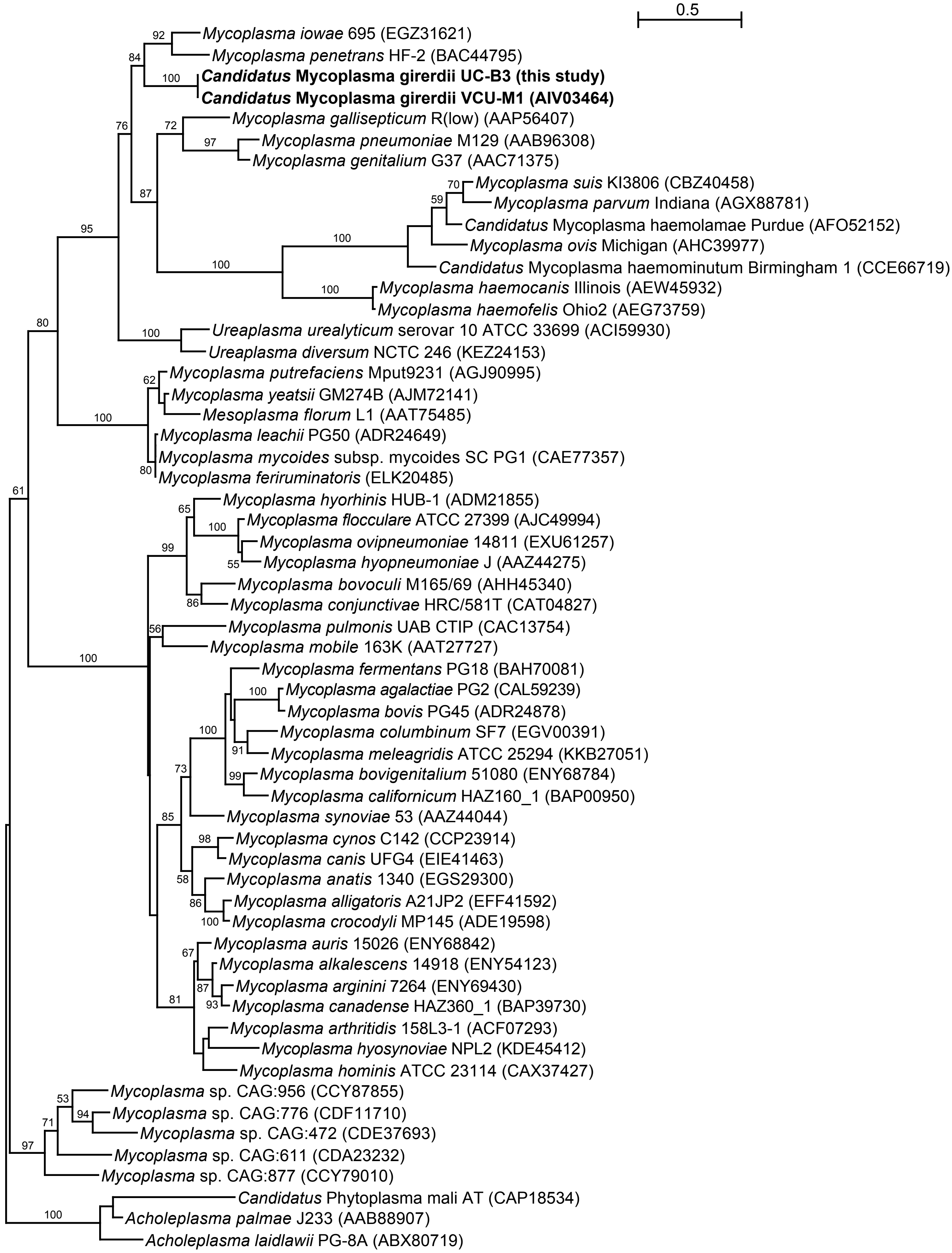

Supplementary Figure S4. Mycoplasma phylogeny including *Ca*. M. girerdii strains. Maximum likelihood phylogeny inferred from an alignment of ribosomal protein S3 amino acid sequences. Bootstrap values > 50% are displayed. The scale bar represents 0.5 substitutions per site. NCBI accession numbers are shown in parentheses.

Figure S4
